# The State of Neurocritical Care Fellowship Training and Attitudes toward Accreditation and Certification: A Survey of Neurocritical Care Fellowship Program Directors

**DOI:** 10.3389/fneur.2017.00548

**Published:** 2017-11-03

**Authors:** Rajat Dhar, Venkatakrishna Rajajee, Anna Finley Caulfield, Matthew B. Maas, Michael L. James, Avinash Bhargava Kumar, Stephen A. Figueroa, David McDonagh, Agnieszka Ardelt

**Affiliations:** ^1^Department of Neurology, Washington University in St. Louis, St. Louis, MO, United States; ^2^Department of Neurology, University of Michigan, Ann Arbor, MI, United States; ^3^Department of Neurosurgery, University of Michigan, Ann Arbor, MI, United States; ^4^Department of Neurology and Neurological Sciences, Stanford University School of Medicine, Stanford, CA, United States; ^5^Department of Neurology, Northwestern University Feinberg School of Medicine, Chicago, IL, United States; ^6^Department of Anesthesiology, Northwestern University Feinberg School of Medicine, Chicago, IL, United States; ^7^Department of Neurology, Duke University Medical Center, Durham, NC, United States; ^8^Department of Anesthesiology, Duke University Medical Center, Durham, NC, United States; ^9^Department of Anesthesiology, Vanderbilt University Medical Center, Nashville, TN, United States; ^10^Department of Critical Care Medicine, Vanderbilt University Medical Center, Nashville, TN, United States; ^11^Department of Neurological Surgery, University of Texas Southwestern, Dallas, TX, United States; ^12^Department of Neurology, University of Texas Southwestern, Dallas, TX, United States; ^13^Department of Anesthesia and Pain Management, University of Texas Southwestern, Dallas, TX, United States; ^14^Department of Neurology (Neurosurgery), University of Chicago, Chicago, IL, United States; ^15^Department of Surgery (Neurosurgery), University of Chicago, Chicago, IL, United States

**Keywords:** neurocritical care, fellowship, training, certification, accreditation

## Abstract

Neurocritical care as a recognized and distinct subspecialty of critical care has grown remarkably since its inception in the 1980s. As of 2016, there were 61 fellowship training programs accredited by the United Council for Neurologic Subspecialties (UCNS) in the United States and more than 1,000 UCNS-certified neurointensivists from diverse medical backgrounds. In late 2015, the Program Accreditation, Physician Certification, and Fellowship Training (PACT) Committee of the Neurocritical Care Society (NCS) was convened to promote and support excellence in the training and certification of neurointensivists. One of the first tasks of the committee was to survey neurocritical care fellowship training program directors to ascertain the current state of fellowship training and attitudes regarding transition to Accreditation Council for Graduate Medical Education (ACGME) accreditation of training programs and American Board of Medical Specialties (ABMS) certification of physicians. First, the survey revealed significant heterogeneities in the manner of neurocritical care training and a lack of consistency in requirements for fellow procedural competency. Second, although a majority of the 33 respondents indicated that a move toward ACGME accreditation/ABMS certification would facilitate further growth and mainstreaming of training in neurocritical care, many programs do not currently meet administrative requirements and do not receive the level of institutional support that would be needed for such a transition. In summary, the results revealed that there is an opportunity for future harmonization of training standards and that a transition to ACGME accreditation/ABMS certification is preferred. While the results reflect the opinions of more than half of the survey respondents, they represent only a small sample of neurointensivists.

## Introduction

Critical care as a dedicated medical subspecialty developed largely because of scientific and technological innovations which allowed the support of patients through catastrophic illness involving organ failure. Neurocritical care as a subspecialty of critical care began in the 1980s as physicians caring for critically ill neurologic patients recognized their unique challenges and formed dedicated intensive care units (ICUs) to optimize their care (1). The Neurocritical Care Society (NCS) was founded in 2002, approximately 20 years after the clinical practice began, and the first annual society meeting was held 1 year later, in 2003 (2). Since then, neurocritical care has grown remarkably: as of 2017, the NCS has over 2,000 members from 50 countries comprising physicians, trainees, nurses, advanced practice providers, and pharmacists (3). Ensuring that a respected and rigorous mechanism exists for certification of physicians in this relatively new field and that future neurointensivists receive high-quality training are cornerstones of the development of the field and acceptance into the mainstream of critical care.

In the United States, accreditation of training programs and certification of physicians are managed by non-governmental, non-profit, self-governed organizations. The Accreditation Council for Graduate Medical Education (ACGME) is the most influential of the training program accrediting bodies, while the member boards of the American Board of Medical Specialties (ABMS) are examples of individual physician certifying bodies. ACGME and ABMS boards require a critical mass of practitioners and specific milestones to confirm that a specialty is clearly defined, recognized, and self-sustaining. Before the 1980s, there was no certification offered in critical care medicine. In September 1980, the ABMS approved the multidisciplinary subspecialty of Critical Care Medicine, and beginning in the late 1980s, individual ABMS member boards provided certification in several critical care subspecialties with overlapping competencies but distinct scopes of practice (4).

Although the foundation of neurocritical care as a valuable independent critical care subspecialty has been propagated by dedicated practitioners for over three decades, accredited training in this field is just completing its first decade. As a relatively new subspecialty, neurocritical care did not initially have the requisite membership and track record to be considered for accreditation and certification through the ACGME–ABMS system but, rather, was developed through the United Council for Neurologic Subspecialties (UCNS). The aim of the UCNS and similar organizations was to organize and structure subspecialties that were not yet prepared for inclusion by the ACGME–ABMS. The UCNS was launched in 2003 with the support of five parent professional organizations representing clinical neuroscience practitioners. The first certificates in neurocritical care were issued in 2007, and fellowship program accreditation followed in 2008.^1^

As of 2017, there were 1,240 UCNS-certified physician neurointensivists with diverse backgrounds including neurology, internal medicine, emergency medicine, and anesthesiology (5). Arguably, neurocritical care in the United States has reached a state of maturity, as training is now offered through 66 UCNS-accredited neurocritical care fellowships (6). In addition to the 2-year fellowship training pathway, a 1-year fellowship is offered by UCNS to neurosurgery residents with at least 4 years of post-graduate clinical training and to fellows who have completed 1 year of post-graduate fellowship training in anesthesiology critical care, surgical critical care, or internal medicine critical care (7). Neurosurgeons also have an alternate pathway to neurocritical care certification through the Committee on Advanced Subspecialty Training (CAST) of the Council of The Society of Neurological Surgeons.^2^

Given the growth and maturation of neurocritical care, accreditation through the ACGME–ABMS pathway is the subject of much discussion among neurointensivists. The Program Accreditation, Physician Certification, and Fellowship Training (PACT) Committee of the NCS was convened to support and promote excellence in training and certification of neurointensivists, and one of the first tasks of the committee was to review the current state of fellowship training. In 2016, a survey was developed by the PACT Committee and e-mailed to fellowship directors to ascertain the level of institutional support, training environment, and challenges faced at this stage of the field’s evolution. The PACT Committee specifically explored how the current UCNS pathway for accreditation of fellowship programs and certification of graduates was perceived and what program directors thought about the transition to the ACGME–ABMS pathway.

## Materials and Methods

Survey questions were compiled from ideas submitted by the members of the PACT Committee and addressed program accreditation, practitioner certification, institutional support, program director responsibilities, faculty and service structure characteristics, trainee characteristics, and training milestones. Respondents were provided opportunities to select categorical answers or numerical entries as well as to enter free-text comments or numbers. Once the committee members were satisfied with survey content, the survey was operationalized using Survey Monkey.^3^ An initial e-mail informing the program directors of the upcoming survey was sent by the NCS administrative office on June 16, 2016; the first e-mail containing the survey was sent to 54 program directors on July 13, 2016; and a reminder e-mail was sent on July 20, 2016. Survey results were analyzed beginning on September 13, 2016.

## Results

Surveys were e-mailed to program directors of 54 of the 57 fellowship programs in existence at the time of the survey, and 33 (61%) of program directors queried completed the surveys. Survey questions and responses are shown in online Supplementary Material.

### Fellowship Accreditation

Thirty-two of 33 (97%) respondents reported UCNS accreditation, while 12 of 31 (39%) reported concomitant CAST accreditation. Among the 12 institutions offering both UCNS-accredited and CAST-accredited neurocritical care fellowships, 2 (17%) had a common program director and 11 (92%) shared faculty.

Of 32 respondents, 22% indicated that neurocritical care not being included in the ACGME–ABMS pathway may adversely affect candidate recruitment, and 35% felt that job opportunities available to graduating fellows may be adversely affected. However, 52% (15/29) felt that the ACGME–ABMS pathway would best facilitate integration of neurocritical care into the critical care mainstream in the future, and 68% (21/31) indicated that ACGME accreditation would be preferred as a vehicle for supporting future growth of neurocritical care as a field. Additionally, 69% (22/32) of respondents similarly indicated that ABMS certification was preferred, while 25% (8/32) preferred the UCNS and 6% (2/32) CAST, for future growth.

### Institutional Support

Approximately half (16/33, 48%) of the responding program directors indicated that they receive institutional support, 30% (10/33) receiving protected time/effort and 18% (6/33) receiving a fixed stipend. The median designated effort reported was 8.5% (IQR 5–10).

Slightly over half of respondents (17/33, 52%) reported having an administrative coordinator with at least a fractional Full Time Equivalent dedicated to the neurocritical care fellowship, but only 18% (6/33) received salary support from the institution for the administrative coordinator.

Approximately three-quarters (25/33, 76%) of programs received institutional support for fellow salaries; one-third (11/33, 33%) utilized clinical revenue for fellow salaries. When institutional support for fellow salaries was provided, all fellows in the program were supported in 20/25 (80%) programs. Among these 20 programs which received salary support for all fellows in the program, the entire salary for each fellow was covered in 15 (75%), and only half of the salary was covered in the remaining 5 (25%). Among programs that received institutional support for fellow salaries, 59% reported that support provided to neurocritical care fellows was not different from that provided to fellows in ACGME-accredited programs at their institution. Three (9%) of 33 directors reported using clinical revenue to support fellows’ research projects.

### Administrative Responsibilities of Program Directors

**Figure 1:**
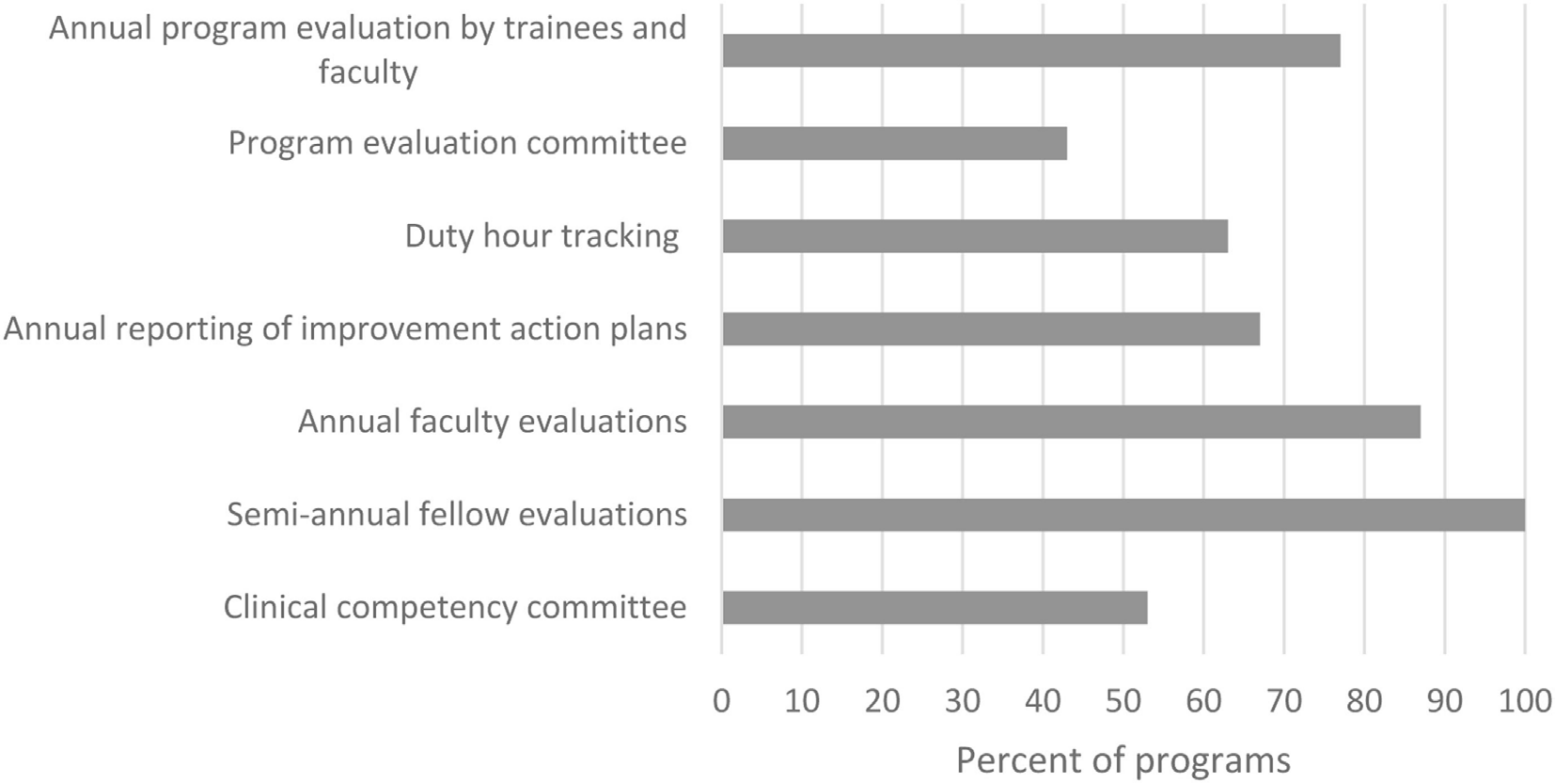
Administrative program requirements reported by 30 fellowship directors.

Fellowship directors were queried about current administrative responsibilities such as would be required of an ACGME-accredited fellowship (Figure 1). While all programs were already completing semi-annual evaluations of their fellows, slightly more than half met other requirements such as having committees for program evaluation and clinical competency. Nonetheless, 69% (22/32) of fellowship directors did not consider fulfilling of all these administrative responsibilities to be unreasonably burdensome.

### Program Faculty

The median number of faculty associated with the training programs surveyed was five; nine programs had eight or more faculty members affiliated with the fellowship, while 18 programs had seven or fewer. Of the 223 faculty members affiliated with fellowships, 149 (67%) were UCNS-certified in neurocritical care. The most common subspecialty affiliation was neurology (68%), followed by anesthesiology (15%), pulmonary/internal medicine (6%), surgery (4%), neurosurgery (3%), and emergency medicine (3%).

### ICU Structure and Coverage Logistics

Sixty percent of respondents characterized their ICUs as “open,” with open units defined as those in which services other than neurocritical care admit patients and enter orders, in contrast to closed units where admission and order entry are under the sole purview of the neurocritical care service.

The number of ICUs covered by the programs’ faculty and fellows ranged from one to seven with the majority covering one (53% of 28) or two (36% of 28). Forty two percent of programs included a step-down unit. In terms of the number of beds covered, the responses ranged from 8 to 54, with a median of 23. The question did not specify the type of beds, ICU or step-down, or whether overflow patients in other patient care areas could be included in the response. Most programs (78%) reported that attending physicians and fellows provided consultations outside of their parent ICU.

**Figure 2:**
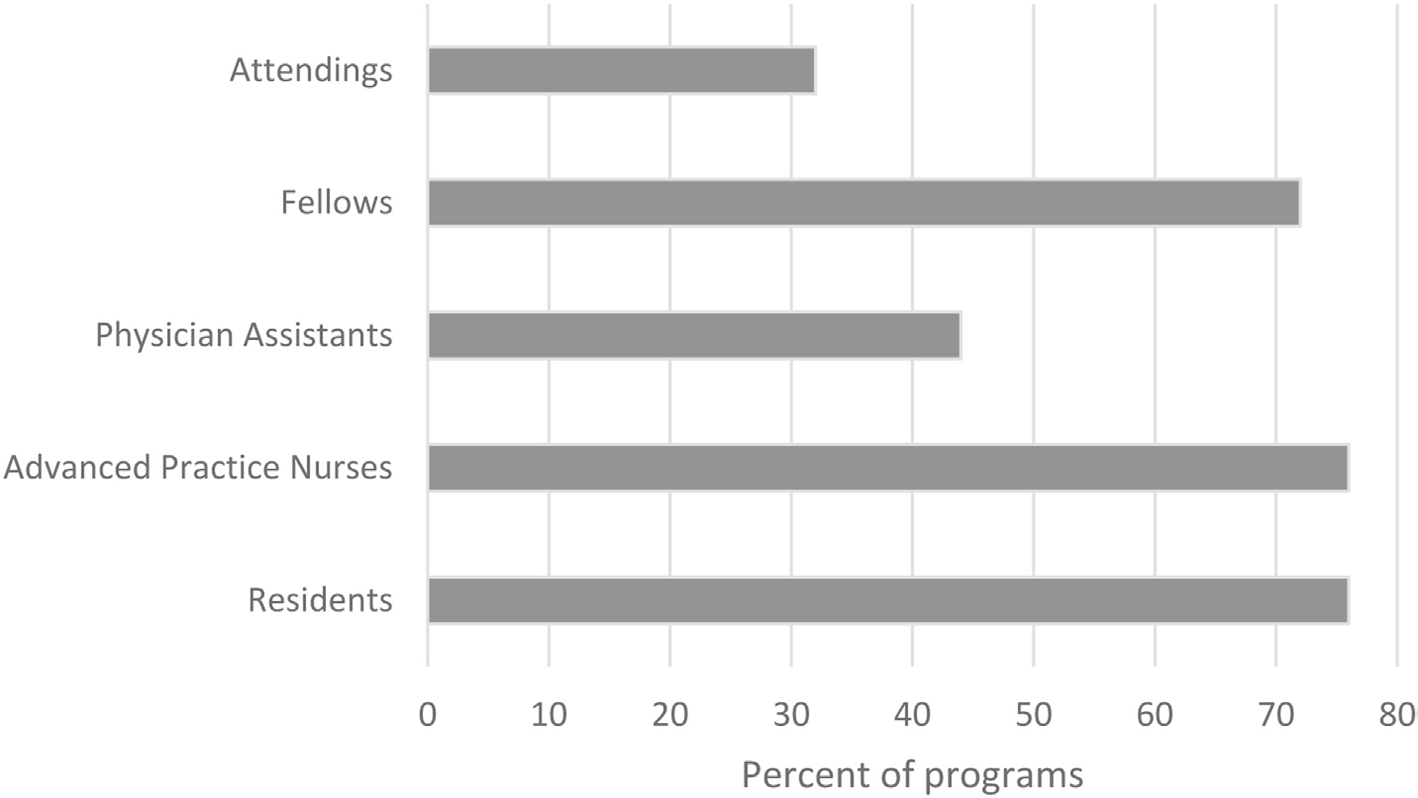
Night-time in-house coverage by provider type reported by 25 respondents.

Ninety-seven percent of programs included residents on the ICU team; 88% included acute care nurse practitioners; and 62% included physician assistants. Of those programs with advanced practice providers, 89% had advanced practice providers who were dedicated to the neurocritical care service. Thirty-two percent of the programs had night-time in-house attending coverage; the remainder had combinations of residents, fellows, and advanced practice providers in-house 24/7 (Figure 2).

### Fellow Recruitment and Characteristics

Nearly all of responding fellowship programs (27/29, 93%) participated in the San Francisco Match system,^4^ although 55% (16/29) reported also offering positions outside of the match. All programs accepted candidates from neurology, and the majority accepted candidates from neurosurgery (25/29, 86%), internal medicine (23/29, 79%), anesthesiology (22/29, 76%), and emergency medicine (21/29, 72%). Two programs (7%) accepted candidates from pediatric neurology and one (3%) from general pediatrics. For the past 3 years, the majority of neurocritical care fellows in training were from the primary specialty of neurology (126 fellows). Internal medicine (15), emergency medicine (7), anesthesia (7), and neurosurgery (2) were less represented.

Sixty-two percent of programs (18/29) required applicants to complete an ACGME or Royal College of Physicians and Surgeons of Canada-accredited residency program. Twenty five of 28 (89%) programs supported J1 visas; 11/28 (39%) H-1B visas; and 9/28 (32%) O-1 visas. Seventy-two percent (21/29) offered 1-year training programs for neurosurgeons and candidates with critical care board eligibility/certification.

Most of the programs (20/28, 71%) credentialed fellows as post-graduate trainees; the remainder credentialed fellows as faculty.

### Fellow Procedure Training and Billing

Most program directors (19/28, 68%) indicated that procedural volumes should be mandated, but there was a wide variation as to procedural requirements and even whether specific procedures were required as part of training (Table 1). All responding program directors required central venous and arterial line placement procedures. Few responding program directors had a required number for bedside tracheostomy and intracranial pressure monitor placement. Approximately one-quarter of responding fellowship programs (7/29, 24%) allowed fellows to independently charge/bill for evaluation and management (E/M) services and procedures.

**Table 1:** Procedural requirements in neurocritical care fellowships.

				Procedural requirements, % respondents				
	Number of respondents	Procedure not required^a^, % respondents	No procedural minimum, % respondents	≤5	10	15	20	≥25
Central venous line	25	0	20	20^b^	40^c^	8	8	4
Arterial line	25	0	24	28^b^	36^c^	0	4	8
Endotracheal intubation	24	8	13	8	17	13^c^	21^b^	21
Thoracentesis	22	14	36	36	14	0	0	0
Paracentesis	21	14	43	29	14	0	0	0
Bronchoscopy	24	21	25	8	33^b^	0	13	0
Bedside tracheostomy	21	62	19	0	0	0	14	5
Critical care ultrasound	21	33	38	5	5	5	10	5
Transcranial Doppler	23	26	30	0	0	0	4	39^d^
Carotid ultrasound	20	45	35	0	0	0	0	20^e^
Lumbar puncture	24	13	42	29^f^	17	0	0	0
Lumbar drain	21	48	29	14	5	5	0	0
Intracranial pressure monitor	21	52	33	0	5	5	5	0
Pulmonary artery catheter	21	19	43	19	19	0	0	0

*The question regarding required minimum volumes for procedural competency appears to have been interpreted in two ways: (1) the minimum necessary for the fellow to complete prior to doing the procedure unsupervised during fellowship or (2) the minimum necessary to complete by the end of fellowship*.

*^a^Not required, not applicable, or to be determined*.

*^b^One respondent reported that this was the number of supervised procedures required before independence*.

*^c^One respondent reported this as the number required per year*.

*^d^One reported 50 required; one required 50 performed and 100 read; and seven programs required 100*.

*^e^One program required 25; three required 100*.

*^f^One indicated 5 was a requirement for fellows without neurology training*.

## Discussion

This is the first comprehensive survey of neurocritical care fellowship training program directors and occurs at a time when changes to accreditation and certification are being pursued. Survey responses provide an overview of the state of training of this maturing field from the point of view of program directors of 33 training programs, which is currently representative of over half of the accredited programs. The main findings are that (1) most program directors favor the ACGME–ABMS pathway as a vehicle for future integration of neurocritical care into mainstream critical care; and (2) there is heterogeneity of institutional structures (open versus closed units, logistics of care provision, and level of fellow independence) and wide variation in procedural requirements among neurocritical care training programs.

Almost all survey respondents directed UCNS-accredited programs, and while UCNS-certification was not thought to be detrimental to fellow recruitment and post-graduate careers, more than half of the respondents indicated that future acceptance and integration of the subspecialty could benefit from ACGME accreditation and ABMS certification. Overall, neurocritical care fellowship programs received less institutional support than comparable fellowships governed by ACGME. While most programs received salary support for fellows, only half received support for the director, and most did not receive support for the administrative coordinator. Per ACGME guidelines, such support would be mandated and would represent a shift from what is currently provided to training programs at many institutions (8). Likewise, ACGME-mandated administrative tasks were already performed in most, but not all, programs. Adherence to these tasks by all programs after transitioning to ACGME accreditation could, therefore, increase costs and administrative burdens, requiring resource shifting or increased resources. There could also be other consequences of a transition to ACGME–ABMS: for example, as faculty-fellows would be disallowed, the change from billing to non-billing fellows might affect the financial viability of some programs. In all, the number of programs able to meet the rigorous ACGME accreditation requirements could be fewer than currently exists under the UCNS system. On the other hand, the number of training programs and fellows may not grow under the UCNS system as many hospitals’ GME offices give credentialing and funding preference to ACGME-accredited programs.

The survey also revealed significant heterogeneity in fellowship training in neurocritical care related to differences in institutional structures as well as wide variation in fellow procedural requirements. While neurology is the major source of fellows, program faculty show a broader representation of backgrounds including a significant number from anesthesiology and other critical care subspecialties. Programs vary in whether they support foreign-trained or visa-sponsored trainees, something that would likely be standardized under ACGME. Although many programs accept candidates into 1-year pathways for neurosurgeons and those with prior critical care training, the percentage of graduates who have completed this track in recent years is unknown.

Fellows train within both open and closed units, and work with residents, advanced practice providers, and faculty. Most coverage models have 24/7 in-house coverage, with fellows as over-night providers in 72%, most often without a night-time attending in-house. Approximately one-quarter of fellows are credentialed as attendings and can bill independently for E/M services and procedures. As previously discussed, with transition to the ACGME–ABMS pathway, revenue in programs where fellows are so credentialed could decline, as independent billing would no longer be permitted.

While the heterogeneous background of faculty, trainees, and the institutional variations complicate the structure of neurocritical care training, they are not unique to neurocritical care. In fact, such heterogeneity is common among the currently recognized ACGME–ABMS disciplines (9).

Variation was also the theme of fellow procedural competency requirements. Although some caution needs to be exercised in the analysis of the results due to different interpretations of “minimum volumes required,” most fellowship directors indicated that there should be specific requirements. Central venous catheter insertion and arterial catheter insertion appeared universally incorporated into fellowship training, but there was significant variability among programs in what was considered a minimum number required for competency. Procedures such as endotracheal intubation, thoracentesis, and intracranial procedures produced an even broader range of responses, from not being required to having various required minimums. These results highlight the current uncertainty around procedural requirements which could potentially lead to variable fellow competency on entry into independent practice. The results, however, also suggest an opportunity to derive consensus about procedural competency in neurocritical care which could lead to future standardization of requirements across training programs (10).

In conclusion, the subspecialty of neurocritical care has transitioned from a few scattered programs accepting and training fellows in an *ad hoc* manner to 66 fellowship training programs currently, most which are formally accredited by the UCNS and, therefore, offer a pathway to UCNS physician certification. The current broad training requirements have allowed many institutions with diverse ICU structures and faculty to match and train fellows in neurocritical care, but given the current maturity level of the subspecialty, an opportunity may exist to standardize some of the training, such as procedural competency. The finding with the greatest potential implications for the subspecialty, however, is that more than half of the survey respondents believe that the ACGME–ABMS pathway is more desirable than the current UCNS pathway going forward. Caution needs to be exercised when interpreting this finding: while this survey represents more than half of neurocritical care fellowship directors, it contains only a small sample of all neurointensivists and may not be reflective of the attitudes of the field as a whole.

## Author Contributions

All authors made substantial contributions to the design of the survey; analysis of the data; drafting, revision, and approval of the manuscript. All authors are accountable for the accuracy and integrity of the work. RD and VR contributed equally to manuscript preparation.

## Conflict of Interest Statement

The authors declare that the research was conducted in the absence of any commercial or financial relationships that could be construed as a potential conflict of interest.

## Footnotes

^1^www.ucns.org.

^2^https://www.societyns.org/fellowships/index.asp.

^3^https://www.surveymonkey.com/.

^4^www.sfmatch.org
