# Supplementary material for: The State of Neurocritical Care Fellowship Training and Attitudes toward Accreditation and Certification: A Survey of Neurocritical Care Fellowship Program Directors
Source: Front Neurol. 2017 Nov 3;8:548. doi: 10.3389/fneur.2017.00548 (PMC5668669; doi:10.3389/fneur.2017.00548)
Supplement: Supplementary file 1 [file Data_Sheet_1.PDF]

Supplementary Material

The State of Neurocritical Care Fellowship Training and Attitudes Toward Accreditation and Certification: A Survey of Neurocritical Care Fellowship Program Directors

Rajat Dhar, Venkatakrishna Rajajee, Anna Finley Caulfield, Matthew Maas, Michael James, Avinash Bhargava Kumar, Stephen A. Figueroa, David McDonagh, Agnieszka Ardelt*

*** Correspondence:** aaardelt@yahoo.com

1 Supplementary Data

Neurocritical care fellowship program directors survey: raw survey responses.

Q1 Is your neurocritical care fellowship program accredited by the UCNS?

Answered: 33 Skipped: 0

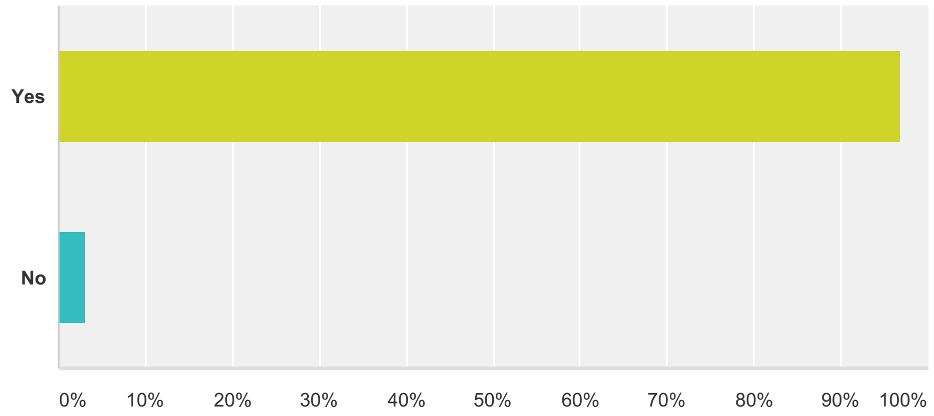

Answer Choices	Responses	
Yes	**96.97%**	32
No	**3.03%**	1
**Total**		**33**

Q2 What forms of institutional program support does your neurocritical care fellowship receive (mark all that apply)?

Answered: 33 Skipped: 0

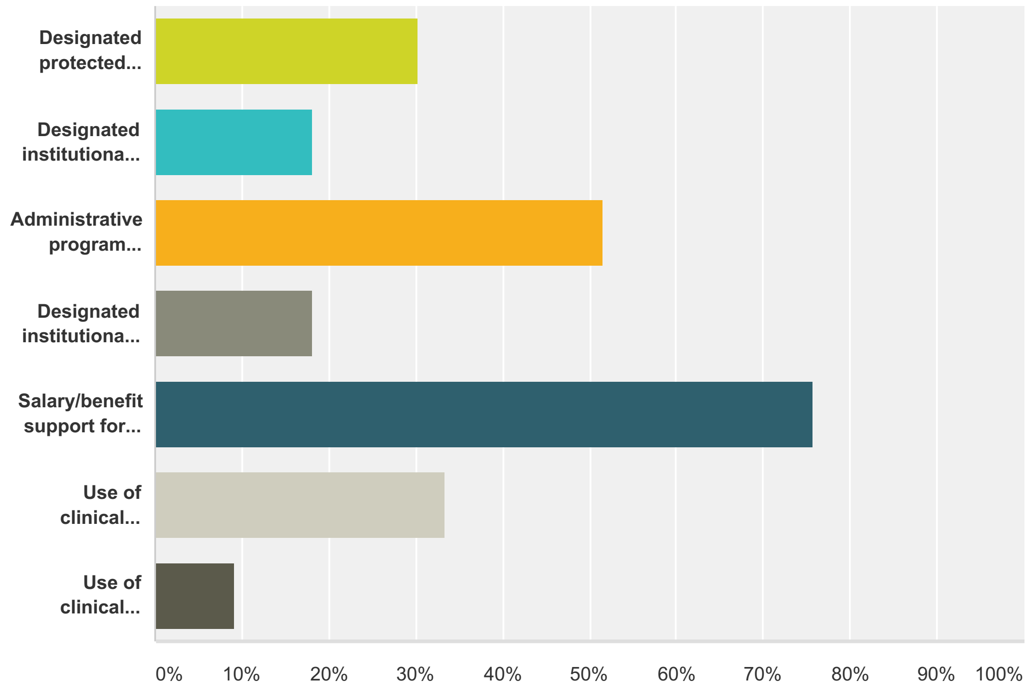

Answer Choices	Responses	
Designated protected time/effort for the neurocritical care fellowship program director. This means a specific percentage of the program director's full time job is set aside for directing the fellowship and is funded institutionally, rather than by grants or clinical work.	**30.30%**	10
Designated institutional stipend for the neurocritical care fellowship program director. This means a specific dollar amount is set aside to compensate the program director for his/her work related to directing the fellowship, regardless of the actual effort expended. The stipend is funded institutionally, rather than by grants or clinical work.	**18.18%**	6
Administrative program coordinator for fellowship. This position must have at least a fractional FTE specifically designated for fellowship program administrative support.	**51.52%**	17
Designated institutional salary support for an administrative program coordinator.	**18.18%**	6
Salary/benefit support for fellows from the institution.	**75.76%**	25
Use of clinical revenue to support fellows' salaries.	**33.33%**	11
Use of clinical revenue to support projects.	**9.09%**	3
**Total Respondents: 33**		

Q3 If there is designated/protected time for the neurocritical care fellowship director, what % effort is protected (please write in)? Those funded by a fixed stipend, please leave this question blank and mark N/A for Question 4.

Answered: 18 Skipped: 15

20% = 1
15% = 1
10% = 2
6% for one fellow, 10% when there are 2 fellows
5% = 5
.1 FTE = 1
.07 FTE = 1
0% = 3
NA = 3
percentage time but no fixed stipend given

Q4 If there is designated/protected time for the neurocritical care fellowship director, is the percent of designated/protected time different than for Accreditation Council for Graduate Medical Education (ACGME) - accredited programs at your institution?

Answered: 29 Skipped: 4

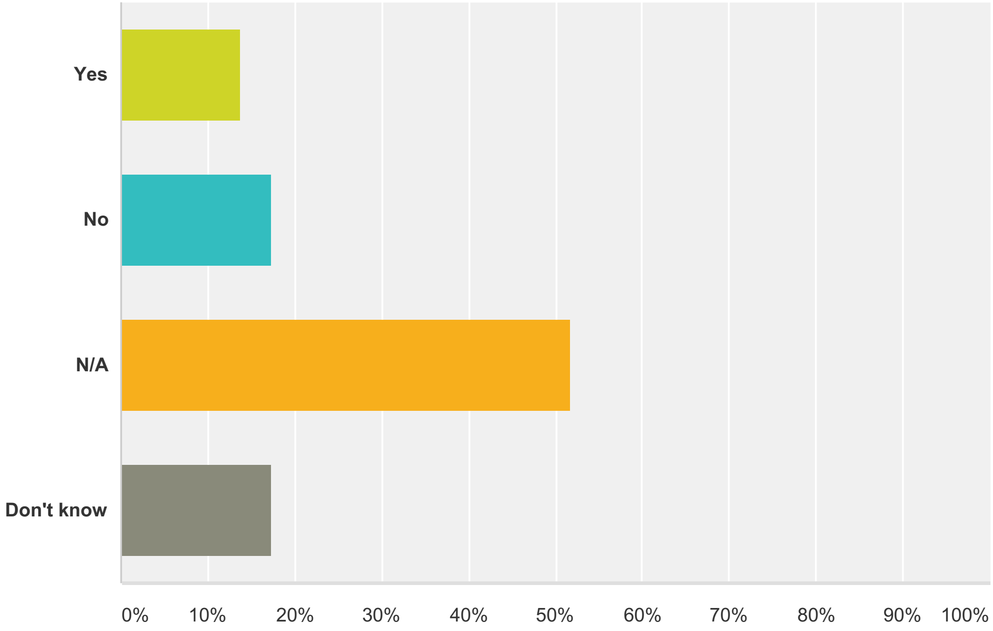

Answer Choices	Responses	
Yes	**13.79%**	4
No	**17.24%**	5
N/A	**51.72%**	15
Don't know	**17.24%**	5
**Total**		**29**

Q5 If there is a designated institutional stipend for the neurocritical care fellowship director, is the amount of the stipend different than for ACGME - accredited programs at your institution? Those funded by percent effort rather than a fixed stipend should mark N/A here.

Answered: 31 Skipped: 2

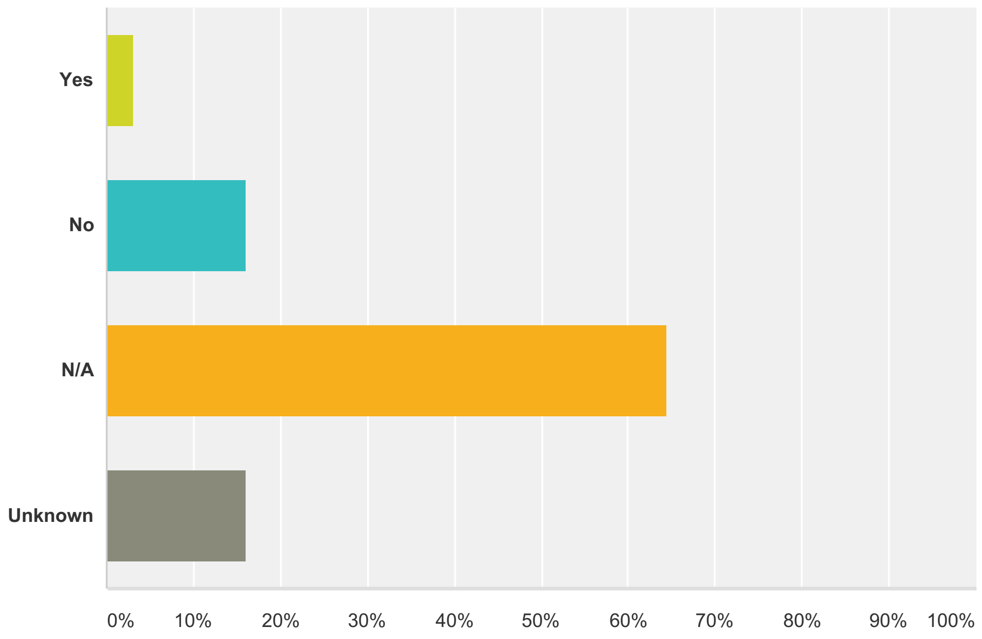

Answer Choices	Responses	
Yes	**3.23%**	1
No	**16.13%**	5
N/A	**64.52%**	20
Unknown	**16.13%**	5
**Total**		**31**

Q6 If there is an administrative program coordinator for the neurocritical care fellowship, is this different than for ACGME - accredited programs at your institution?

Answered: 29 Skipped: 4

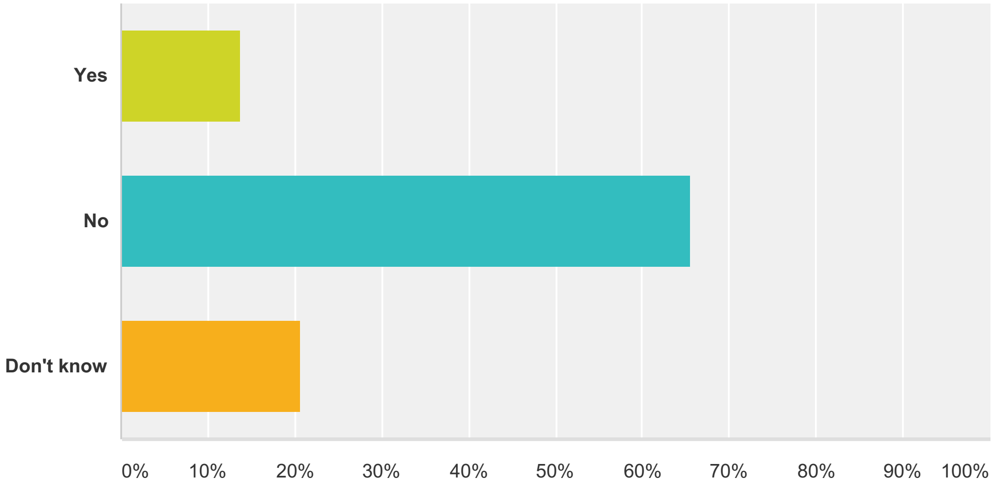

Answer Choices	Responses	
Yes	**13.79%**	4
No	**65.52%**	19
Don't know	**20.69%**	6
**Total**		**29**

Q7 If there is designated institutional salary support for the neurocritical care fellowship administrative program coordinator, what % effort is institutionally supported (please write in)?

Answered: 13 Skipped: 20

#	Responses	Date
1	N/A	7/22/2016 10:34 AM
2	n/a	7/22/2016 8:12 AM
3	this comes from clinical income	7/20/2016 7:47 PM
4	100%	7/20/2016 2:34 PM
5	It's not specified, but rather "fellowship" activities are listed as part of her job description, which I am told is not unlike other subspecialities with small fellowship programs at our institution.	7/15/2016 6:15 PM
6	I don't know the percentage	7/14/2016 3:43 PM
7	None	7/14/2016 11:05 AM
8	30%	7/13/2016 9:59 PM
9	None	7/13/2016 6:02 PM
10	Na	7/13/2016 5:48 PM
11	I don't know	7/13/2016 5:23 PM
12	don't know	7/13/2016 4:54 PM
13	NA	7/13/2016 4:29 PM

Q8 If there is designated institutional salary support for the neurocritical care fellowship administrative program coordinator, is this different than for ACGME - accredited programs at your institution?

Answered: 26 Skipped: 7

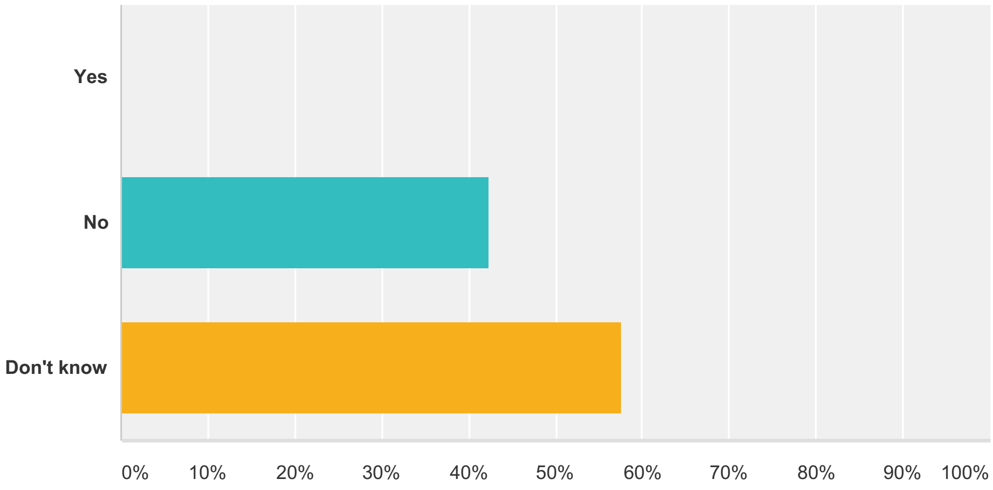

Answer Choices	Responses	
Yes	**0.00%**	0
No	**42.31%**	11
Don't know	**57.69%**	15
**Total**		**26**

Q9 If there is salary/ benefit support for neurocritical care fellows from the institution, how many fellows receive institutional support (please write how many out of the total)?

Answered: 26 Skipped: 7

#	Responses	Date
1	4 out of 4	7/22/2016 1:03 PM
2	7/7	7/22/2016 10:34 AM
3	2/4	7/22/2016 8:12 AM
4	1	7/21/2016 12:46 PM
5	None	7/20/2016 7:47 PM
6	4 of 4	7/20/2016 5:12 PM
7	4 (all)	7/20/2016 3:34 PM
8	all two of them per year	7/20/2016 3:13 PM
9	4/4	7/20/2016 2:34 PM
10	7	7/20/2016 9:24 AM
11	2/2 (1 per year for 2 year accredited fellowship) First year funded by hospital Second year funded through Vascular neurology ACGME fellowship slot and they have to meet Vascular Neurology requirements in addition to UCNS NCC requirements.	7/19/2016 12:45 PM
12	9/9	7/18/2016 5:07 PM
13	All	7/15/2016 6:15 PM
14	6/6	7/14/2016 5:19 PM
15	4 of 5/year	7/14/2016 3:43 PM
16	1	7/14/2016 11:05 AM
17	2 out of 2	7/13/2016 9:59 PM
18	4 fellows	7/13/2016 9:41 PM
19	9	7/13/2016 8:03 PM
20	None	7/13/2016 6:02 PM
21	3 of 4	7/13/2016 5:48 PM
22	They receive salary, benefits, and trip / travel reimbursement for presentations, talks, and basic memberships	7/13/2016 5:23 PM
23	2/2	7/13/2016 4:54 PM
24	One	7/13/2016 4:38 PM
25	1	7/13/2016 4:29 PM
26	5/5	6/22/2016 11:18 AM

Q10 If there is salary/ benefit support for neurocritical care fellows from the institution, what % FTE is supported (please write in)?

Answered: 23 Skipped: 10

#	Responses	Date
1	50%	7/22/2016 1:03 PM
2	100	7/22/2016 10:34 AM
3	1.0	7/22/2016 8:12 AM
4	1.0	7/21/2016 12:46 PM
5	None	7/20/2016 7:47 PM
6	Hospital 100%	7/20/2016 5:12 PM
7	100%	7/20/2016 3:34 PM
8	100%	7/20/2016 2:34 PM
9	100	7/20/2016 9:24 AM
10	100%	7/19/2016 12:45 PM
11	They are fully supported by the institution	7/18/2016 5:07 PM
12	100%	7/15/2016 6:15 PM
13	100% of the 4. 0% of the 5th	7/14/2016 3:43 PM
14	0.5	7/14/2016 11:05 AM
15	100%	7/13/2016 9:59 PM
16	100%	7/13/2016 9:41 PM
17	100%	7/13/2016 8:03 PM
18	None	7/13/2016 6:02 PM
19	Full fte for 3 of 4 fellows	7/13/2016 5:48 PM
20	I don't know	7/13/2016 5:23 PM
21	100	7/13/2016 4:54 PM
22	50 percent	7/13/2016 4:38 PM
23	100%	6/22/2016 11:18 AM

Q11 If there is salary/ benefit support for neurocritical care fellows from the institution, is this different than for ACGME - accredited programs at your institution?

Answered: 29 Skipped: 4

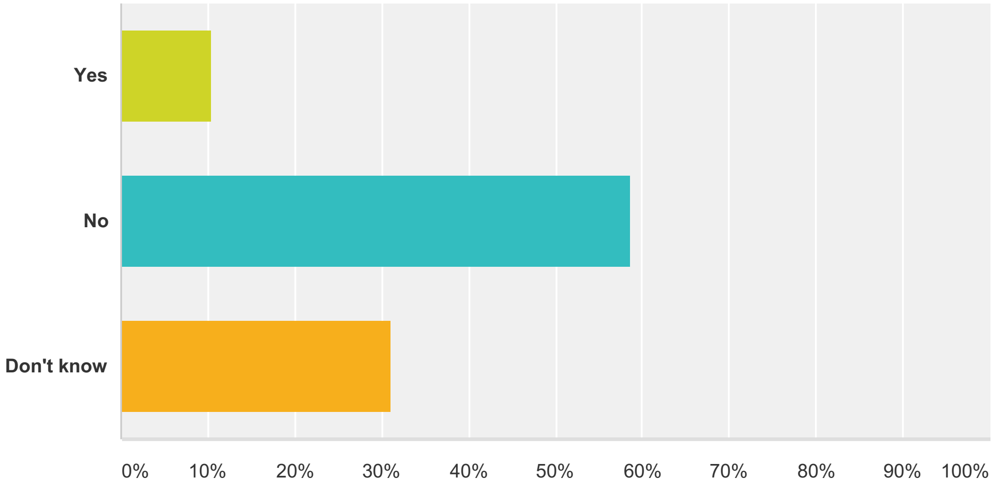

Answer Choices	Responses	
Yes	**10.34%**	3
No	**58.62%**	17
Don't know	**31.03%**	9
**Total**		**29**

Q12 With which of the following policies and procedures currently required of ACGME - accredited programs does your neurocritical care fellowship program currently have to comply (mark all that apply)?

Answered: 30 Skipped: 3

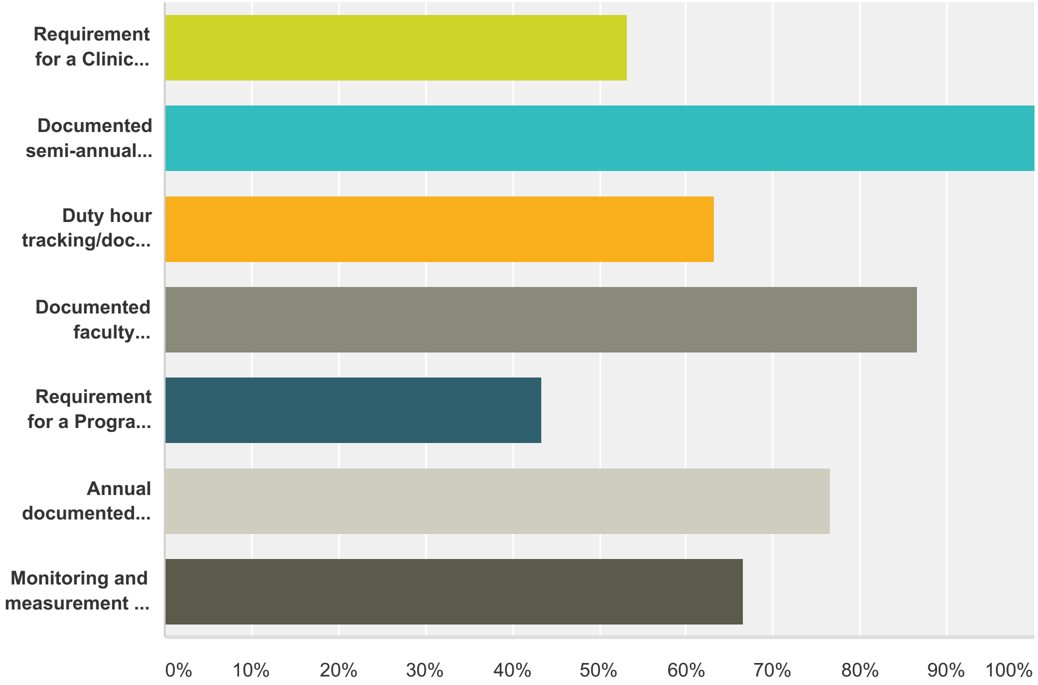

Answer Choices	Responses	
Requirement for a Clinical Competency Committee for fellow evaluations	53.33%	16
Documented semi-annual fellow evaluations	100.00%	30
Duty hour tracking/documentation	63.33%	19
Documented faculty evaluations, at least annually	86.67%	26
Requirement for a Program Evaluation Committee (PEC)	43.33%	13
Annual documented program evaluations using written feedback from trainees and faculty (Note: the ACGME requires annual reporting in the areas of fellow performance, faculty development and progress on previous years' action plans)	76.67%	23
Monitoring and measurement of trainee and faculty performance/development with annual reporting of action plans for improvement	66.67%	20
Total Respondents: 30		

Q13 Do you think the policy and procedural requirements of ACGME accreditation will impose an unreasonable burden on your fellowship program?

Answered: 32 Skipped: 1

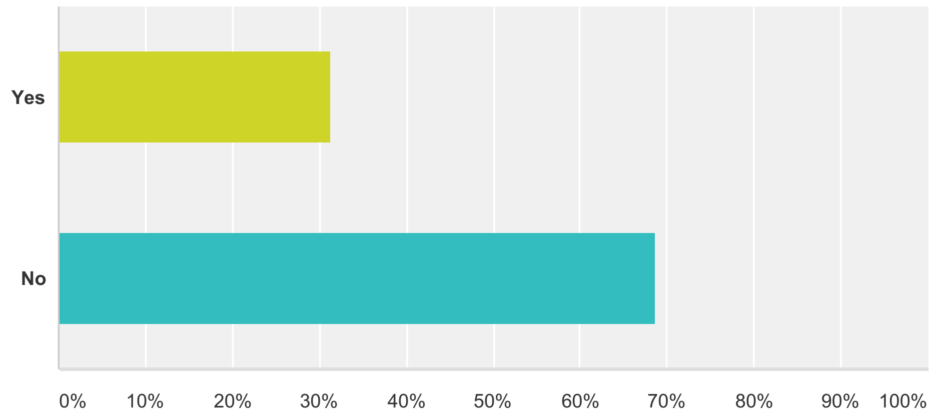

Answer Choices	Responses	
Yes	**31.25%**	10
No	**68.75%**	22
**Total**		**32**

Q14 Do you believe the accreditation system for neurocritical care (UCNS instead of ACGME) has negatively affected your program's ability to recruit excellent candidates?

Answered: 32 Skipped: 1

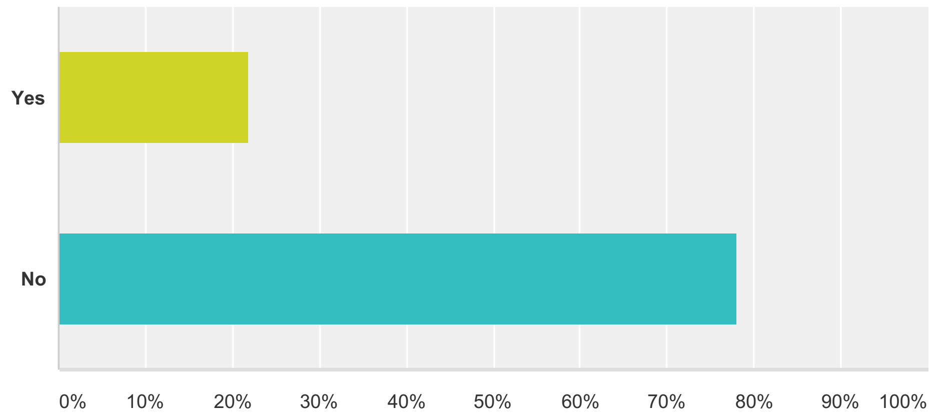

Answer Choices	Responses	
Yes	**21.88%**	7
No	**78.13%**	25
**Total**		**32**

Q15 Does the source of accreditation and certification for neurocritical care through a non - ACGME or non - American Board of Medical Specialties (ABMS) system negatively influence job opportunities available to your graduating fellows?

Answered: 32 Skipped: 1

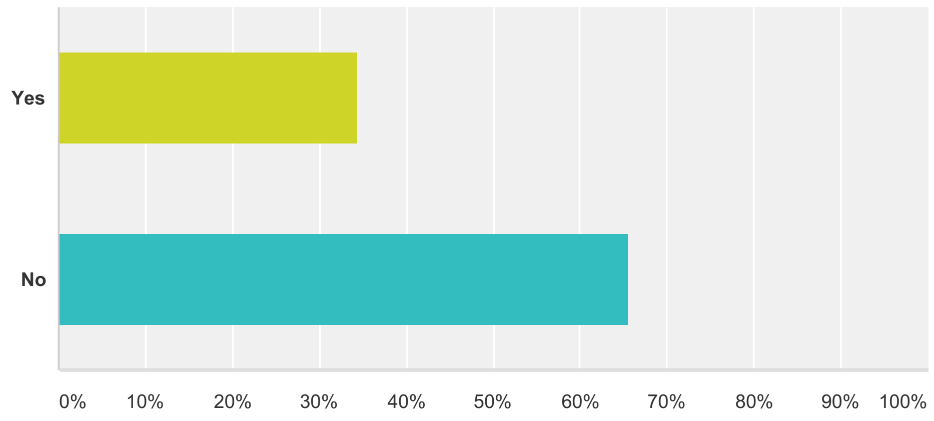

Answer Choices	Responses	
Yes	**34.38%**	11
No	**65.63%**	21
**Total**		**32**

Q16 Future integration of neurointensivists into general critical care training and certification pathways would be best facilitated by (in other words, what would best facilitate "mainstreaming" of NCC into the critical care world so that we could participate in joint certification pathways with medical/anesthesiology/surgical intensivists?)

Answered: 29 Skipped: 4

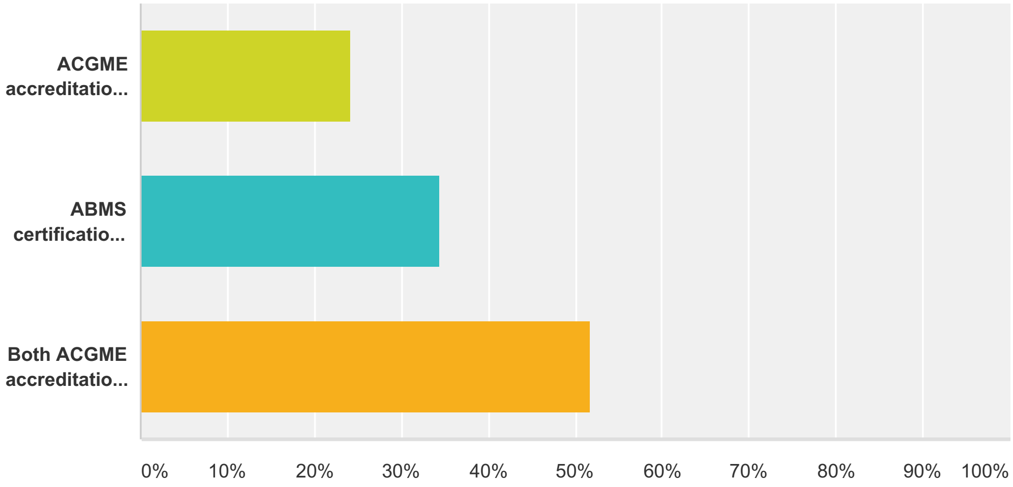

Answer Choices	Responses	
ACGME accreditation of neurocritical care training	**24.14%**	7
ABMS certification of neurointensivists	**34.48%**	10
Both ACGME accreditation and ABMS certification	**51.72%**	15
**Total Respondents: 29**		

Q17 What is the best administrative structure for ACCREDITATION in order to support the growth of neurocritical care as a field?

Answered: 31 Skipped: 2

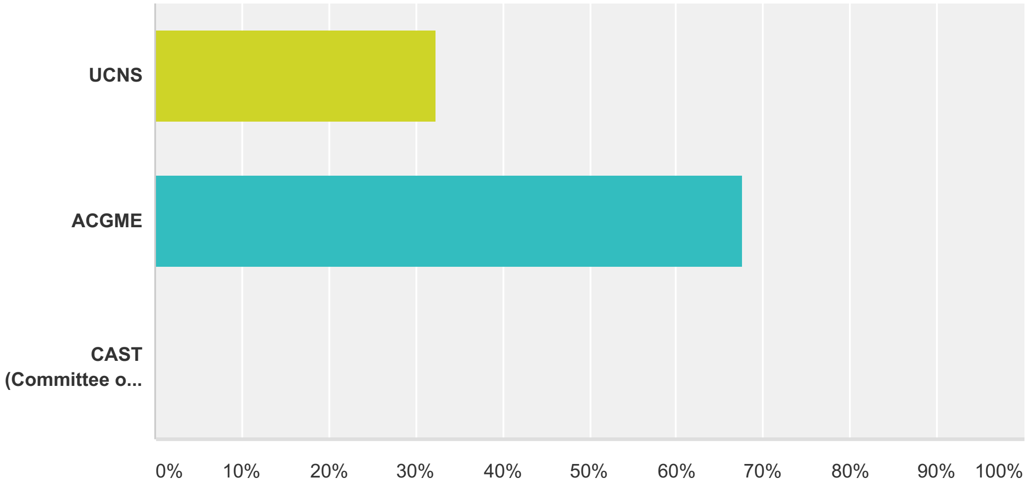

Answer Choices	Responses	
UCNS	**32.26%**	10
ACGME	**67.74%**	21
CAST (Committee on Subspecialty Training)	**0.00%**	0
**Total**		**31**

Q18 What is the best administrative structure for CERTIFICATION in order to support the growth of neurocritical care as a field?

Answered: 32 Skipped: 1

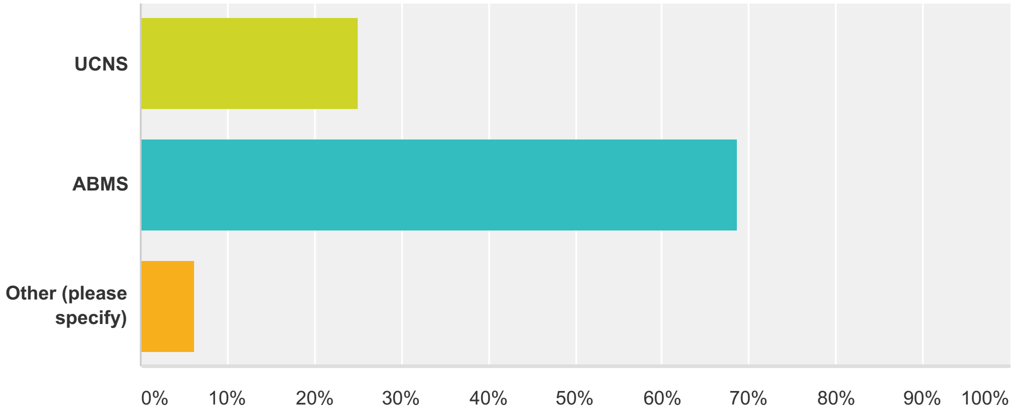

Answer Choices	Responses	
UCNS	**25.00%**	8
ABMS	**68.75%**	22
Other (please specify)	**6.25%**	2
**Total**		**32**

Q19 Does your institution offer a CAST - accredited neurocritical care fellowship?

Answered: 29 Skipped: 4

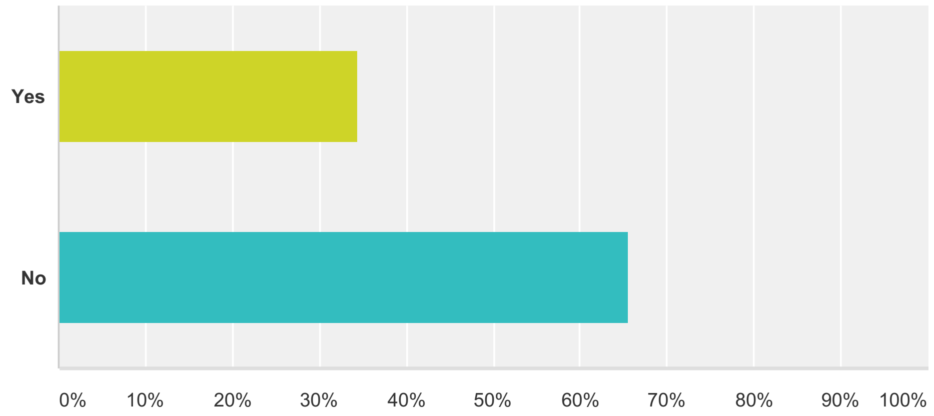

Answer Choices	Responses	
Yes	**34.48%**	10
No	**65.52%**	19
**Total**		**29**

Q20 For institutions with both UCNS - accredited and CAST - accredited neurocritical care fellowship programs, do the programs have the same program director?

Answered: 29   Skipped: 4

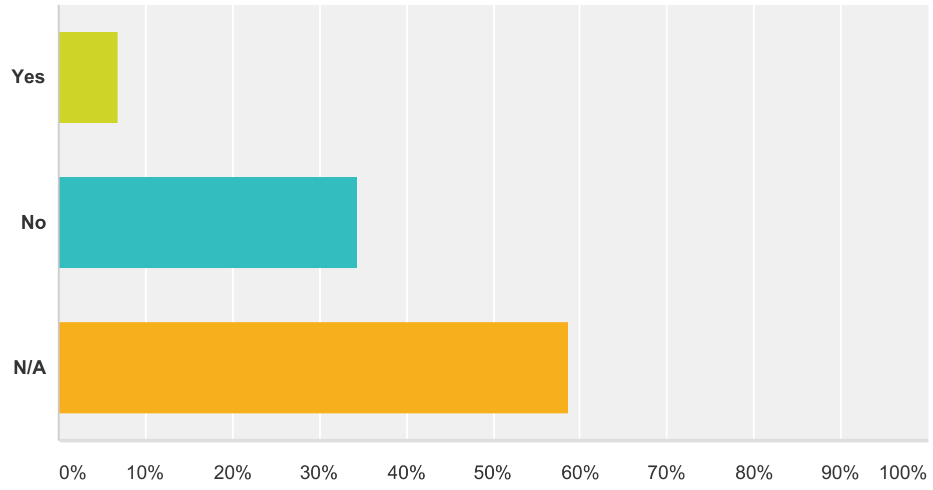

Answer Choices	Responses	
Yes	**6.90%**	2
No	**34.48%**	10
N/A	**58.62%**	17
**Total**		**29**

Q21 For institutions with both UCNS - accredited and CAST - accredited neurocritical care fellowship programs, do the programs have the same faculty?

Answered: 28 Skipped: 5

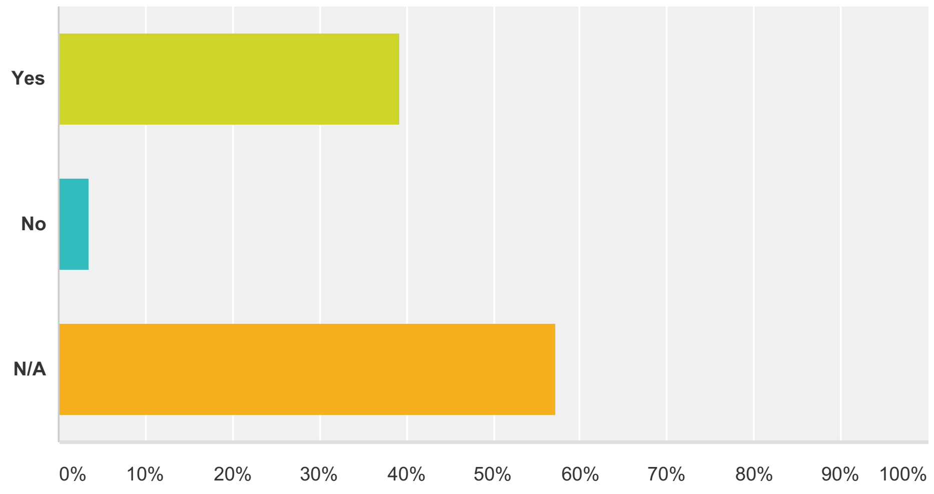

Answer Choices	Responses	
Yes	**39.29%**	11
No	**3.57%**	1
N/A	**57.14%**	16
**Total**		**28**

Q22 Does your neurocritical care fellowship program participate in the San Francisco (SF) Match?

Answered: 29 Skipped: 4

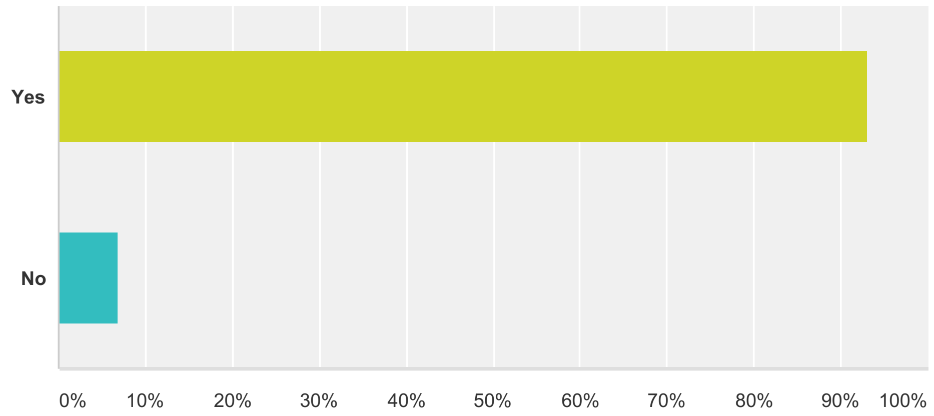

Answer Choices	Responses	
Yes	**93.10%**	27
No	**6.90%**	2
**Total**		**29**

Q23 Does your neurocritical care fellowship program ever offer positions outside of the SF Match?

Answered: 29 Skipped: 4

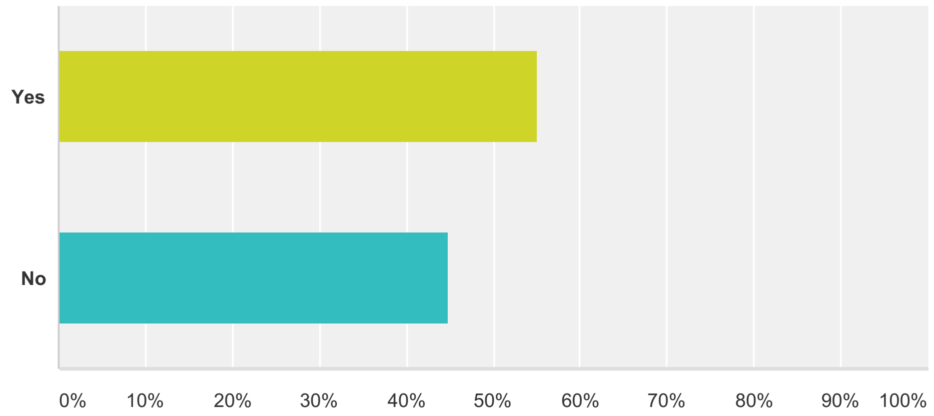

Answer Choices	Responses	
Yes	**55.17%**	16
No	**44.83%**	13
**Total**		**29**

Q24 From which specialties does your neurocritical care program accept candidates for training (mark all that apply)?

Answered: 29 Skipped: 4

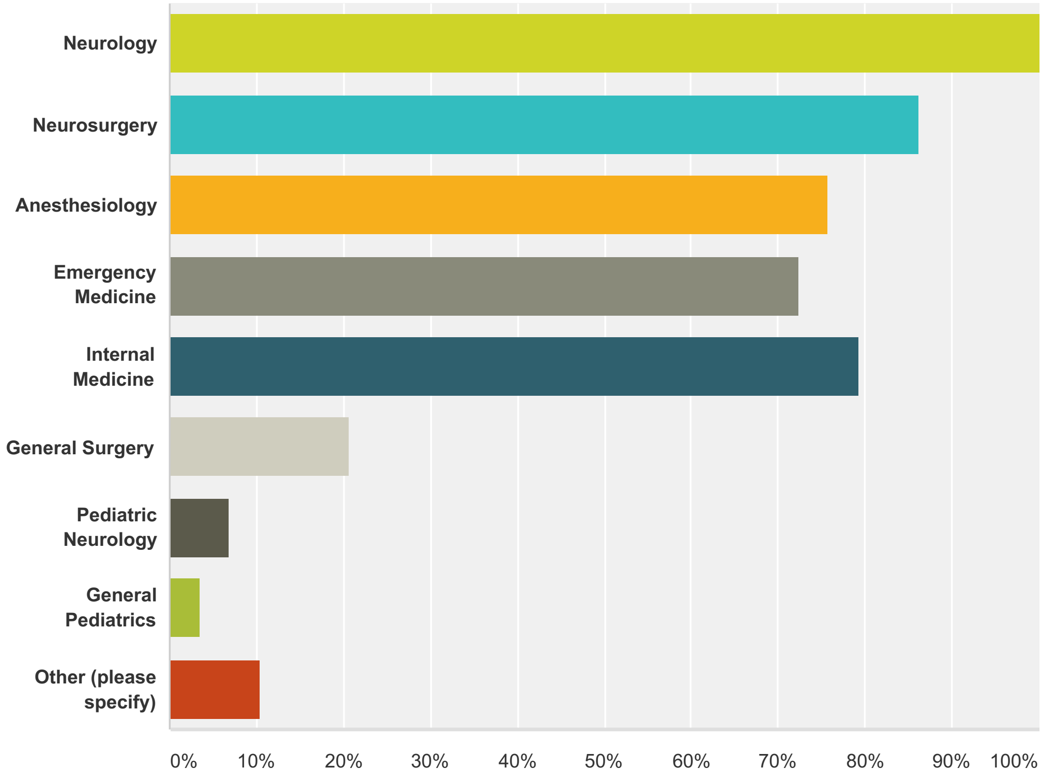

Answer Choices	Responses	
Neurology	**100.00%**	29
Neurosurgery	**86.21%**	25
Anesthesiology	**75.86%**	22
Emergency Medicine	**72.41%**	21
Internal Medicine	**79.31%**	23
General Surgery	**20.69%**	6
Pediatric Neurology	**6.90%**	2
General Pediatrics	**3.45%**	1
Other (please specify)	**10.34%**	3
**Total Respondents: 29**		

Q25 How many fellows from each one of the specialties below have trained/are training in your neurocritical care fellowship program during the past three years (please write in)?

Answered: 29 Skipped: 4

Answer Choices	Responses	
Neurology	**96.55%**	28
Neurosurgery	**34.48%**	10
Anesthesiology	**34.48%**	10
Emergency Medicine	**37.93%**	11
Internal Medicine	**51.72%**	15
General Surgery	**13.79%**	4
Pediatric Neurology	**13.79%**	4
General Pediatrics	**13.79%**	4
Other	**10.34%**	3

#	Neurology	Date
1	3	7/22/2016 3:04 PM
2	6	7/22/2016 1:06 PM
3	10	7/22/2016 10:42 AM
4	6	7/22/2016 8:20 AM
5	1	7/21/2016 12:49 PM
6	5	7/21/2016 9:20 AM
7	4	7/21/2016 3:55 AM
8	6	7/20/2016 7:49 PM
9	6	7/20/2016 5:14 PM
10	5	7/20/2016 3:39 PM
11	3	7/20/2016 3:17 PM
12	1	7/20/2016 2:37 PM
13	5	7/20/2016 1:25 PM
14	13	7/20/2016 9:41 AM
15	6	7/15/2016 6:22 PM
16	1	7/15/2016 7:03 AM
17	6	7/14/2016 3:46 PM
18	3	7/13/2016 10:11 PM
19	5	7/13/2016 9:45 PM
20	8	7/13/2016 8:11 PM

21	2	7/13/2016 6:04 PM
22	3	7/13/2016 5:55 PM
23	2	7/13/2016 5:26 PM
24	3	7/13/2016 5:22 PM
25	5	7/13/2016 5:03 PM
26	0	7/13/2016 4:40 PM
27	2	7/13/2016 4:32 PM
28	6	6/22/2016 11:21 AM

#	Neurosurgery	Date
1	0	7/22/2016 1:06 PM
2	0	7/21/2016 9:20 AM
3	0	7/20/2016 7:49 PM
4	0	7/20/2016 5:14 PM
5	0	7/20/2016 1:25 PM
6	0	7/14/2016 3:46 PM
7	1	7/13/2016 9:45 PM
8	0	7/13/2016 8:11 PM
9	1	7/13/2016 5:55 PM
10	0	7/13/2016 4:40 PM

#	Anesthesiology	Date
1	0	7/22/2016 1:06 PM
2	1	7/21/2016 12:49 PM
3	0	7/21/2016 9:20 AM
4	1	7/20/2016 5:14 PM
5	1	7/20/2016 3:39 PM
6	2	7/20/2016 1:25 PM
7	0	7/14/2016 3:46 PM
8	1	7/13/2016 8:11 PM
9	1	7/13/2016 5:55 PM
10	0	7/13/2016 4:40 PM

#	Emergency Medicine	Date
1	0	7/22/2016 1:06 PM
2	1	7/22/2016 10:42 AM
3	1	7/22/2016 8:20 AM
4	0	7/21/2016 12:49 PM
5	0	7/21/2016 9:20 AM
6	1	7/21/2016 3:55 AM
7	1	7/20/2016 2:37 PM
8	0	7/14/2016 3:46 PM
9	2	7/13/2016 10:11 PM
10	1	7/13/2016 8:11 PM

11	0	7/13/2016 4:40 PM
**#**	**Internal Medicine**	**Date**
1	0	7/22/2016 1:06 PM
2	2	7/22/2016 10:42 AM
3	0	7/21/2016 9:20 AM
4	2	7/21/2016 3:55 AM
5	1	7/20/2016 7:49 PM
6	1	7/20/2016 3:39 PM
7	1	7/20/2016 3:17 PM
8	1	7/20/2016 1:25 PM
9	1	7/20/2016 9:41 AM
10	0	7/14/2016 3:46 PM
11	1	7/14/2016 11:10 AM
12	1	7/13/2016 8:11 PM
13	2	7/13/2016 5:55 PM
14	0	7/13/2016 4:40 PM
15	2	6/22/2016 11:21 AM
**#**	**General Surgery**	**Date**
1	0	7/22/2016 1:06 PM
2	0	7/21/2016 9:20 AM
3	0	7/14/2016 3:46 PM
4	0	7/13/2016 4:40 PM
**#**	**Pediatric Neurology**	**Date**
1	0	7/22/2016 1:06 PM
2	0	7/21/2016 9:20 AM
3	0	7/14/2016 3:46 PM
4	0	7/13/2016 4:40 PM
**#**	**General Pediatrics**	**Date**
1	0	7/22/2016 1:06 PM
2	0	7/21/2016 9:20 AM
3	0	7/14/2016 3:46 PM
4	0	7/13/2016 4:40 PM
**#**	**Other**	**Date**
1	0	7/22/2016 1:06 PM
2	0	7/14/2016 3:46 PM
3	0	7/13/2016 4:40 PM

Q26 Does your neurocritical care fellowship program offer a one - year training pathway for candidates with critical care board eligibility / certification?

Answered: 29 Skipped: 4

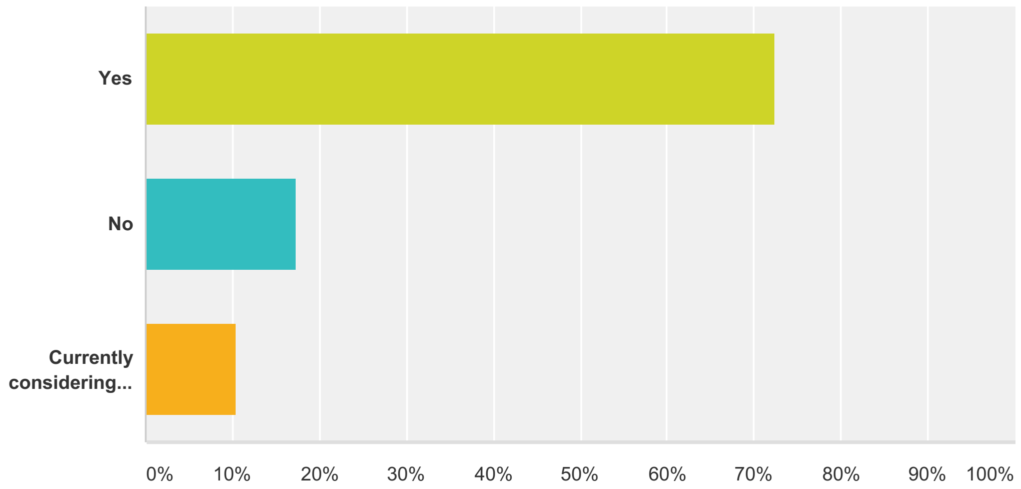

Answer Choices	Responses	
Yes	**72.41%**	21
No	**17.24%**	5
Currently considering offering	**10.34%**	3
**Total**		**29**

Q27 Does your neurocritical care fellowship program offer a one - year training pathway for candidates with the requisite neurosurgical training?

Answered: 29 Skipped: 4

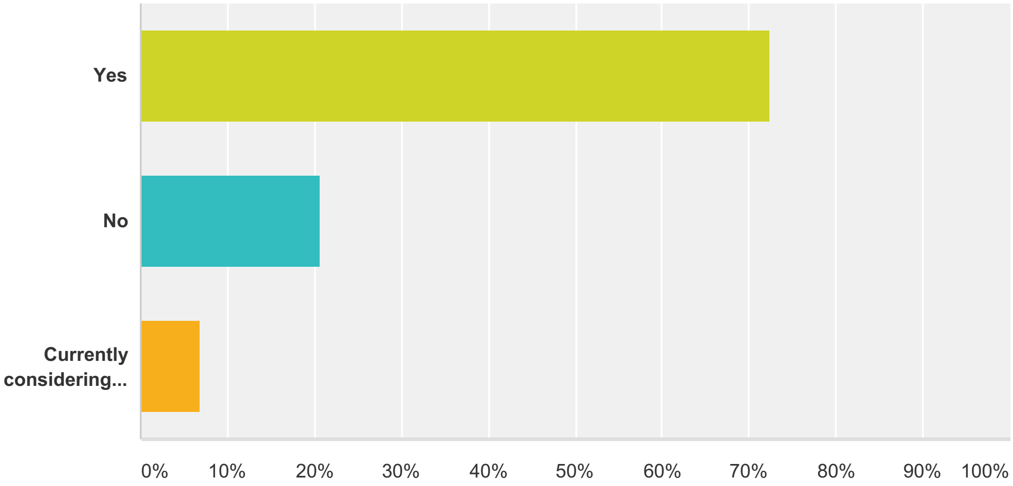

Answer Choices	Responses	
Yes	**72.41%**	21
No	**20.69%**	6
Currently considering offering	**6.90%**	2
**Total**		**29**

Q28 Which of the following visas does your institution sponsor for international medical graduates in the neurocritical care fellowship (mark all that apply)?

Answered: 28 Skipped: 5

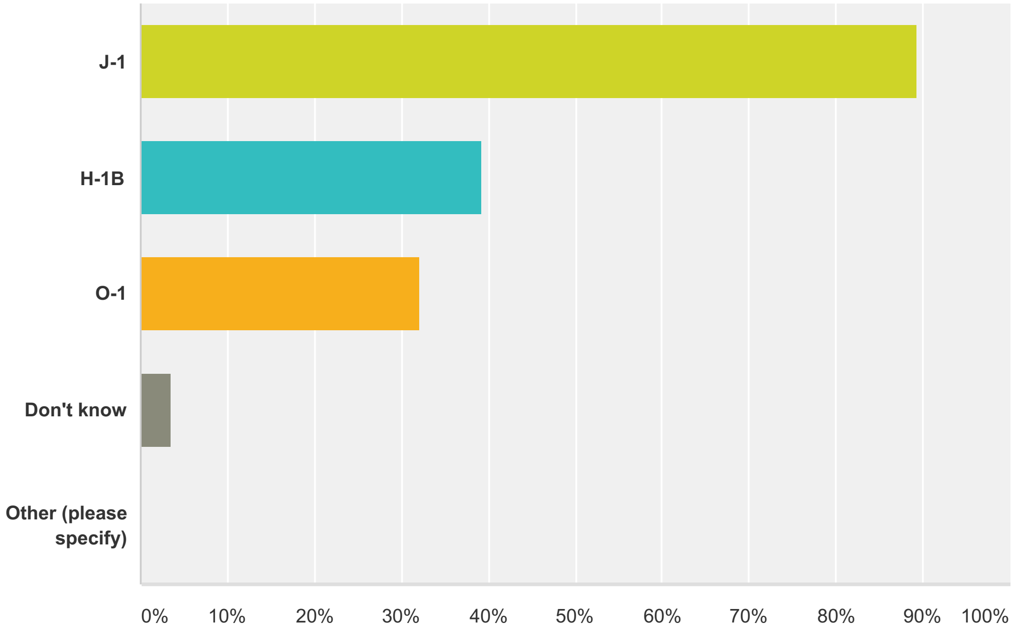

Answer Choices	Responses	
J-1	89.29%	25
H-1B	39.29%	11
O-1	32.14%	9
Don't know	3.57%	1
Other (please specify)	0.00%	0
Total Respondents: 28		

Q29 Do you accept candidates for training in your neurocritical care fellowship who did not finish residency in North America?

Answered: 29 Skipped: 4

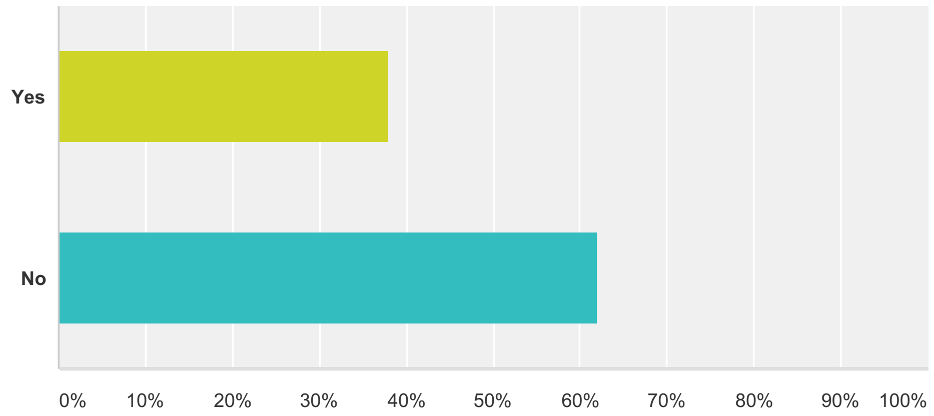

Answer Choices	Responses	
Yes	**37.93%**	11
No	**62.07%**	18
**Total**		**29**

Q30 How are the neurocritical care fellows at your institution credentialed (mark all that apply)?

Answered: 28 Skipped: 5

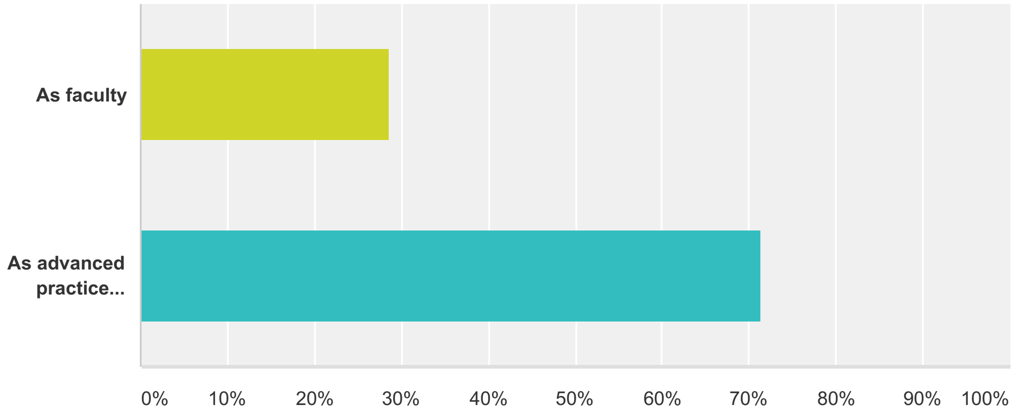

Answer Choices	Responses	
As faculty	28.57%	8
As advanced practice trainees/ non - ACGME accredited post-graduate trainees through the GME office	71.43%	20
**Total**		**28**

Q31 Are your fellows currently allowed to independently bill for E/M services?

Answered: 29 Skipped: 4

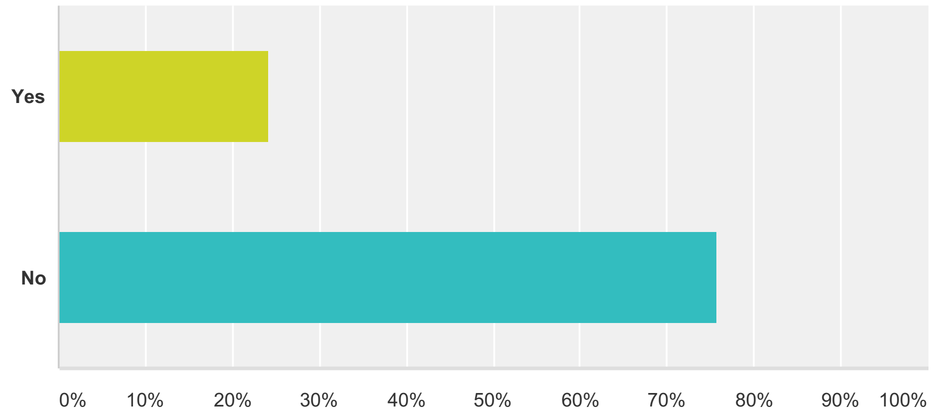

Answer Choices	Responses	
Yes	**24.14%**	7
No	**75.86%**	22
**Total**		**29**

Q32 Are your fellows currently allowed to independently bill for procedures?

Answered: 29 Skipped: 4

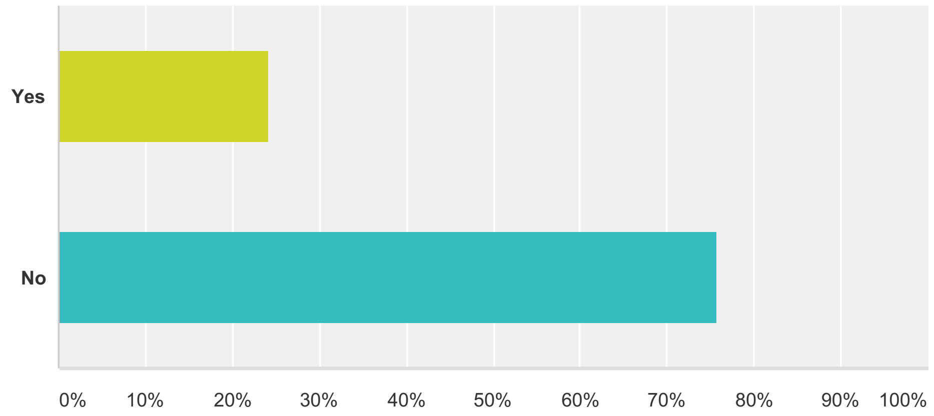

Answer Choices	Responses	
Yes	**24.14%**	7
No	**75.86%**	22
**Total**		**29**

Q33 Does your neurocritical care program have required minimum volumes for procedural competency for fellows (fill in the required number of procedures; type "no minimum" or "not required" if there is no minimum or the procedure is not required, respectively)?

Answered: 25 Skipped: 8

Answer Choices	Responses	
Central venous line	**100.00%**	25
Arterial line	**100.00%**	25
Endotracheal intubation	**96.00%**	24
Thoracentesis	**88.00%**	22
Paracentesis	**84.00%**	21
Bronchoscopy	**96.00%**	24
Bedside tracheostomy	**84.00%**	21
Critical care ultrasound	**84.00%**	21
Transcranial Doppler	**88.00%**	22
Carotid ultrasound	**80.00%**	20
Lumbar puncture	**96.00%**	24
Lumbar drain	**84.00%**	21
Intracranial pressure monitor	**84.00%**	21
Pulmonary artery catheter	**84.00%**	21

#	Central venous line	Date
1	10	7/22/2016 1:06 PM
2	No minimum	7/22/2016 10:42 AM
3	at least 10 independent per year	7/22/2016 8:20 AM
4	4	7/21/2016 9:20 AM
5	10	7/21/2016 3:55 AM
6	No minimim	7/20/2016 5:14 PM
7	10	7/20/2016 3:39 PM
8	20	7/20/2016 3:17 PM
9	10	7/20/2016 2:37 PM
10	30	7/20/2016 1:25 PM
11	10	7/20/2016 9:41 AM
12	Fellow has to do 5 supervised before able to do on own)	7/15/2016 6:22 PM

13	10	7/15/2016 7:03 AM
14	10	7/14/2016 3:46 PM
15	10	7/14/2016 11:10 AM
16	15 (5 IJ, 5 SC, 5 fem)	7/13/2016 10:11 PM
17	15	7/13/2016 8:11 PM
18	No minimum	7/13/2016 6:04 PM
19	5	7/13/2016 5:55 PM
20	10	7/13/2016 5:26 PM
21	3	7/13/2016 5:22 PM
22	no minimum	7/13/2016 5:03 PM
23	5	7/13/2016 4:40 PM
24	no minimum	7/13/2016 4:32 PM
25	20	6/22/2016 11:21 AM

#	Arterial line	Date
1	5	7/22/2016 1:06 PM
2	No minimum	7/22/2016 10:42 AM
3	at least 10 independent per year	7/22/2016 8:20 AM
4	4	7/21/2016 9:20 AM
5	10	7/21/2016 3:55 AM
6	No minimim	7/20/2016 5:14 PM
7	10	7/20/2016 3:39 PM
8	20	7/20/2016 3:17 PM
9	10	7/20/2016 2:37 PM
10	30	7/20/2016 1:25 PM
11	10	7/20/2016 9:41 AM
12	same as above	7/15/2016 6:22 PM
13	5	7/15/2016 7:03 AM
14	10	7/14/2016 3:46 PM
15	30	7/14/2016 11:10 AM
16	10	7/13/2016 10:11 PM
17	5	7/13/2016 8:11 PM
18	No minimum	7/13/2016 6:04 PM
19	5	7/13/2016 5:55 PM
20	10	7/13/2016 5:26 PM
21	3	7/13/2016 5:22 PM
22	no minimum	7/13/2016 5:03 PM
23	No	7/13/2016 4:40 PM
24	no minimum	7/13/2016 4:32 PM
25	10	6/22/2016 11:21 AM

#	Endotracheal intubation	Date
1	20	7/22/2016 1:06 PM

2	40	7/22/2016 10:42 AM
3	15 per year	7/22/2016 8:20 AM
4	4	7/21/2016 9:20 AM
5	20	7/21/2016 3:55 AM
6	not required	7/20/2016 3:39 PM
7	20	7/20/2016 3:17 PM
8	10	7/20/2016 2:37 PM
9	30	7/20/2016 1:25 PM
10	15	7/20/2016 9:41 AM
11	Fellow has to do 20 supervised before able to do on own)	7/15/2016 6:22 PM
12	10	7/15/2016 7:03 AM
13	25	7/14/2016 3:46 PM
14	30	7/14/2016 11:10 AM
15	15	7/13/2016 10:11 PM
16	not required	7/13/2016 8:11 PM
17	No minimum	7/13/2016 6:04 PM
18	10	7/13/2016 5:55 PM
19	20	7/13/2016 5:26 PM
20	10	7/13/2016 5:22 PM
21	no minimum	7/13/2016 5:03 PM
22	5	7/13/2016 4:40 PM
23	no minimum	7/13/2016 4:32 PM
24	50	6/22/2016 11:21 AM

#	Thoracentesis	Date
1	no minimum	7/22/2016 1:06 PM
2	no minimum	7/22/2016 8:20 AM
3	4	7/21/2016 9:20 AM
4	5	7/21/2016 3:55 AM
5	not required	7/20/2016 3:39 PM
6	5	7/20/2016 3:17 PM
7	no minimum	7/20/2016 2:37 PM
8	10	7/20/2016 9:41 AM
9	no minimum	7/15/2016 6:22 PM
10	5	7/15/2016 7:03 AM
11	10	7/14/2016 3:46 PM
12	no minimum	7/14/2016 11:10 AM
13	5	7/13/2016 10:11 PM
14	5	7/13/2016 8:11 PM
15	No minimum	7/13/2016 6:04 PM
16	Not required	7/13/2016 5:55 PM
17	10	7/13/2016 5:26 PM

18	3	7/13/2016 5:22 PM
19	no minimum	7/13/2016 5:03 PM
20	5	7/13/2016 4:40 PM
21	no minimum	7/13/2016 4:32 PM
22	N/A	6/22/2016 11:21 AM

#	Paracentesis	Date
1	no minimum	7/22/2016 1:06 PM
2	no minimum	7/22/2016 8:20 AM
3	4	7/21/2016 9:20 AM
4	no minimum	7/21/2016 3:55 AM
5	not required	7/20/2016 3:39 PM
6	no minimum	7/20/2016 2:37 PM
7	10	7/20/2016 9:41 AM
8	no minimum	7/15/2016 6:22 PM
9	5	7/15/2016 7:03 AM
10	10	7/14/2016 3:46 PM
11	no minimum	7/14/2016 11:10 AM
12	3	7/13/2016 10:11 PM
13	5	7/13/2016 8:11 PM
14	No minimum	7/13/2016 6:04 PM
15	Not required	7/13/2016 5:55 PM
16	10	7/13/2016 5:26 PM
17	3	7/13/2016 5:22 PM
18	no minimum	7/13/2016 5:03 PM
19	5	7/13/2016 4:40 PM
20	no minimum	7/13/2016 4:32 PM
21	N/A	6/22/2016 11:21 AM

#	Bronchoscopy	Date
1	not required	7/22/2016 1:06 PM
2	No minimum	7/22/2016 10:42 AM
3	no minimum	7/22/2016 8:20 AM
4	not required	7/21/2016 9:20 AM
5	10	7/21/2016 3:55 AM
6	10	7/20/2016 3:39 PM
7	10	7/20/2016 3:17 PM
8	10	7/20/2016 2:37 PM
9	10	7/20/2016 1:25 PM
10	10	7/20/2016 9:41 AM
11	Fellow has to do 10 supervised before able to do on own	7/15/2016 6:22 PM
12	not required	7/15/2016 7:03 AM
13	20	7/14/2016 3:46 PM

14	no minimum	7/14/2016 11:10 AM
15	10	7/13/2016 10:11 PM
16	5	7/13/2016 8:11 PM
17	No minimum	7/13/2016 6:04 PM
18	Not required	7/13/2016 5:55 PM
19	20	7/13/2016 5:26 PM
20	5	7/13/2016 5:22 PM
21	no minimum	7/13/2016 5:03 PM
22	to be determined	7/13/2016 4:40 PM
23	no minimum	7/13/2016 4:32 PM
24	20	6/22/2016 11:21 AM

#	Bedside tracheostomy	Date
1	not required	7/22/2016 1:06 PM
2	do not do	7/22/2016 8:20 AM
3	not required	7/21/2016 9:20 AM
4	20	7/21/2016 3:55 AM
5	not required	7/20/2016 3:39 PM
6	not required	7/20/2016 2:37 PM
7	20	7/20/2016 9:41 AM
8	no minimum	7/15/2016 6:22 PM
9	not required	7/15/2016 7:03 AM
10	na	7/14/2016 3:46 PM
11	no minimum	7/14/2016 11:10 AM
12	not required	7/13/2016 10:11 PM
13	not required	7/13/2016 8:11 PM
14	No minimum	7/13/2016 6:04 PM
15	Not required	7/13/2016 5:55 PM
16	20	7/13/2016 5:26 PM
17	25	7/13/2016 5:22 PM
18	not required	7/13/2016 5:03 PM
19	not required	7/13/2016 4:40 PM
20	no minimum	7/13/2016 4:32 PM
21	N/A	6/22/2016 11:21 AM

#	Critical care ultrasound	Date
1	no minimum	7/22/2016 1:06 PM
2	at least 15 per year	7/22/2016 8:20 AM
3	not required	7/21/2016 9:20 AM
4	20	7/21/2016 3:55 AM
5	no minimum	7/20/2016 3:39 PM
6	no minimum	7/20/2016 2:37 PM
7	10	7/20/2016 9:41 AM

8	we are working on minimum for this	7/15/2016 6:22 PM
9	5	7/15/2016 7:03 AM
10	na	7/14/2016 3:46 PM
11	no minimum	7/14/2016 11:10 AM
12	50	7/13/2016 10:11 PM
13	not required	7/13/2016 8:11 PM
14	No minimum	7/13/2016 6:04 PM
15	Not required	7/13/2016 5:55 PM
16	20	7/13/2016 5:26 PM
17	no min	7/13/2016 5:22 PM
18	no minimum	7/13/2016 5:03 PM
19	to be determined	7/13/2016 4:40 PM
20	no minimum	7/13/2016 4:32 PM
21	N/A	6/22/2016 11:21 AM

#	Transcranial Doppler	Date
1	not required	7/22/2016 1:06 PM
2	perform 50; read 100	7/22/2016 8:20 AM
3	not required	7/21/2016 9:20 AM
4	100	7/21/2016 3:55 AM
5	100	7/20/2016 3:39 PM
6	20	7/20/2016 3:17 PM
7	no minimum	7/20/2016 2:37 PM
8	100 - for credentialing	7/20/2016 9:41 AM
9	we are working on minimum for this	7/15/2016 6:22 PM
10	not required	7/15/2016 7:03 AM
11	100	7/14/2016 3:46 PM
12	no minimum	7/14/2016 11:10 AM
13	no minimum	7/13/2016 10:11 PM
14	not required	7/13/2016 8:11 PM
15	No minimum	7/13/2016 6:04 PM
16	As per certification requirements	7/13/2016 5:55 PM
17	100	7/13/2016 5:26 PM
18	no min	7/13/2016 5:22 PM
19	no minimum	7/13/2016 5:03 PM
20	to be determined	7/13/2016 4:40 PM
21	no minimum	7/13/2016 4:32 PM
22	50	6/22/2016 11:21 AM

#	Carotid ultrasound	Date
1	not required	7/22/2016 1:06 PM
2	do not do	7/22/2016 8:20 AM
3	not required	7/21/2016 9:20 AM

4	no minimum	7/21/2016 3:55 AM
5	100	7/20/2016 3:39 PM
6	not required	7/20/2016 2:37 PM
7	100 - for credentialing	7/20/2016 9:41 AM
8	no minimum	7/15/2016 6:22 PM
9	not required	7/15/2016 7:03 AM
10	100	7/14/2016 3:46 PM
11	no minimum	7/14/2016 11:10 AM
12	not required	7/13/2016 10:11 PM
13	not required	7/13/2016 8:11 PM
14	No minimum	7/13/2016 6:04 PM
15	Not required	7/13/2016 5:55 PM
16	no min	7/13/2016 5:22 PM
17	no minimum	7/13/2016 5:03 PM
18	not required	7/13/2016 4:40 PM
19	no minimum	7/13/2016 4:32 PM
20	25	6/22/2016 11:21 AM

#	Lumbar puncture	Date
1	no minimum	7/22/2016 1:06 PM
2	No minimum	7/22/2016 10:42 AM
3	no minimum	7/22/2016 8:20 AM
4	4	7/21/2016 9:20 AM
5	no minimum	7/21/2016 3:55 AM
6	no minimum	7/20/2016 3:39 PM
7	10	7/20/2016 3:17 PM
8	10	7/20/2016 2:37 PM
9	10	7/20/2016 1:25 PM
10	10	7/20/2016 9:41 AM
11	no minimum	7/15/2016 6:22 PM
12	no minimum	7/15/2016 7:03 AM
13	NA	7/14/2016 3:46 PM
14	5	7/14/2016 11:10 AM
15	3	7/13/2016 10:11 PM
16	5	7/13/2016 8:11 PM
17	No minimum	7/13/2016 6:04 PM
18	If no neurology 5	7/13/2016 5:55 PM
19	5	7/13/2016 5:26 PM
20	3	7/13/2016 5:22 PM
21	no minimum	7/13/2016 5:03 PM
22	NO	7/13/2016 4:40 PM
23	no minimum	7/13/2016 4:32 PM

24	N/A	6/22/2016 11:21 AM
**#**	**Lumbar drain**	**Date**
1	not required	7/22/2016 1:06 PM
2	no minimum	7/22/2016 8:20 AM
3	not required	7/21/2016 9:20 AM
4	10	7/21/2016 3:55 AM
5	not required	7/20/2016 3:39 PM
6	not required	7/20/2016 2:37 PM
7	15	7/20/2016 9:41 AM
8	no minimum	7/15/2016 6:22 PM
9	5	7/15/2016 7:03 AM
10	NA	7/14/2016 3:46 PM
11	no minimum	7/14/2016 11:10 AM
12	not required	7/13/2016 10:11 PM
13	not required	7/13/2016 8:11 PM
14	No minimum	7/13/2016 6:04 PM
15	Not required	7/13/2016 5:55 PM
16	5	7/13/2016 5:26 PM
17	3	7/13/2016 5:22 PM
18	no minimum	7/13/2016 5:03 PM
19	not required	7/13/2016 4:40 PM
20	no minimum	7/13/2016 4:32 PM
21	N/A	6/22/2016 11:21 AM
**#**	**Intracranial pressure monitor**	**Date**
1	not required	7/22/2016 1:06 PM
2	no minimum	7/22/2016 8:20 AM
3	not required	7/21/2016 9:20 AM
4	no minimum	7/21/2016 3:55 AM
5	not required	7/20/2016 3:39 PM
6	not required	7/20/2016 2:37 PM
7	15	7/20/2016 9:41 AM
8	no minimum	7/15/2016 6:22 PM
9	not required	7/15/2016 7:03 AM
10	NA	7/14/2016 3:46 PM
11	no minimum	7/14/2016 11:10 AM
12	not required	7/13/2016 10:11 PM
13	not required	7/13/2016 8:11 PM
14	No minimum	7/13/2016 6:04 PM
15	Not required	7/13/2016 5:55 PM
16	20	7/13/2016 5:26 PM
17	10	7/13/2016 5:22 PM

18	no minimum	7/13/2016 5:03 PM
19	to be determined	7/13/2016 4:40 PM
20	no minimum	7/13/2016 4:32 PM
21	N/A	6/22/2016 11:21 AM

#	Pulmonary artery catheter	Date
1	no minimum	7/22/2016 1:06 PM
2	no minimum	7/22/2016 8:20 AM
3	4	7/21/2016 9:20 AM
4	no minimum	7/21/2016 3:55 AM
5	10	7/20/2016 3:39 PM
6	no minimum	7/20/2016 2:37 PM
7	10	7/20/2016 9:41 AM
8	no minimum	7/15/2016 6:22 PM
9	not required	7/15/2016 7:03 AM
10	10	7/14/2016 3:46 PM
11	no minimum	7/14/2016 11:10 AM
12	2	7/13/2016 10:11 PM
13	not required	7/13/2016 8:11 PM
14	No minimum	7/13/2016 6:04 PM
15	5	7/13/2016 5:55 PM
16	10	7/13/2016 5:26 PM
17	3	7/13/2016 5:22 PM
18	no minimum	7/13/2016 5:03 PM
19	to be determined	7/13/2016 4:40 PM
20	no minimum	7/13/2016 4:32 PM
21	N/A	6/22/2016 11:21 AM

Q34 Should procedural volumes be mandated for all neurocritical care training fellowships?

Answered: 28 Skipped: 5

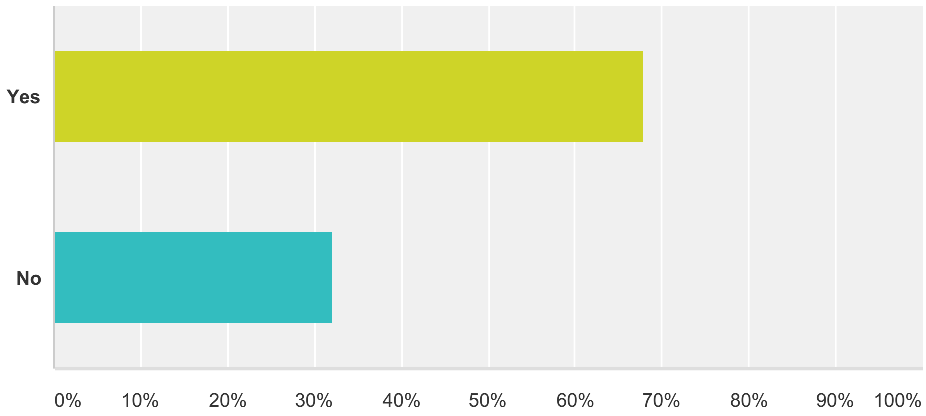

Answer Choices	Responses	
Yes	**67.86%**	19
No	**32.14%**	9
**Total**		**28**

Q35 How many faculty are there in your neurocritical care fellowship program?

Answered: 28 Skipped: 5

#	Responses	Date
1	~ 50 critical care faculty	7/22/2016 3:04 PM
2	5	7/22/2016 1:06 PM
3	8	7/22/2016 10:42 AM
4	4	7/22/2016 8:20 AM
5	4	7/21/2016 12:49 PM
6	4	7/21/2016 9:20 AM
7	5	7/21/2016 3:55 AM
8	5	7/20/2016 5:14 PM
9	8	7/20/2016 3:39 PM
10	3	7/20/2016 3:17 PM
11	3	7/20/2016 2:37 PM
12	7	7/20/2016 1:25 PM
13	8	7/20/2016 9:41 AM
14	6	7/15/2016 6:22 PM
15	3	7/15/2016 7:03 AM
16	8	7/14/2016 3:46 PM
17	13	7/14/2016 11:10 AM
18	12	7/13/2016 10:11 PM
19	15	7/13/2016 9:45 PM
20	10	7/13/2016 8:11 PM
21	5	7/13/2016 6:04 PM
22	4	7/13/2016 5:55 PM
23	5	7/13/2016 5:26 PM
24	5	7/13/2016 5:22 PM
25	4	7/13/2016 5:03 PM
26	four	7/13/2016 4:40 PM
27	6	7/13/2016 4:32 PM
28	8	6/22/2016 11:21 AM

Q36 Of the faculty in your neurocritical care fellowship training program, how many are UCNS - certified (please write in)?

Answered: 28 Skipped: 5

#	Responses	Date
1	5	7/22/2016 3:04 PM
2	4	7/22/2016 1:06 PM
3	6	7/22/2016 10:42 AM
4	4	7/22/2016 8:20 AM
5	2	7/21/2016 12:49 PM
6	3	7/21/2016 9:20 AM
7	5	7/21/2016 3:55 AM
8	5	7/20/2016 5:14 PM
9	8	7/20/2016 3:39 PM
10	2	7/20/2016 3:17 PM
11	3	7/20/2016 2:37 PM
12	6	7/20/2016 1:25 PM
13	6	7/20/2016 9:41 AM
14	6	7/15/2016 6:22 PM
15	3	7/15/2016 7:03 AM
16	all	7/14/2016 3:46 PM
17	4	7/14/2016 11:10 AM
18	8	7/13/2016 10:11 PM
19	15	7/13/2016 9:45 PM
20	10	7/13/2016 8:11 PM
21	4	7/13/2016 6:04 PM
22	All 4/4	7/13/2016 5:55 PM
23	5	7/13/2016 5:26 PM
24	3	7/13/2016 5:22 PM
25	3	7/13/2016 5:03 PM
26	all	7/13/2016 4:40 PM
27	5	7/13/2016 4:32 PM
28	8	6/22/2016 11:21 AM

Q37 Of the faculty in your neurocritical care fellowship program, how many are from the following subspecialties (please write in the number; leave blank if not applicable)?

Answered: 27 Skipped: 6

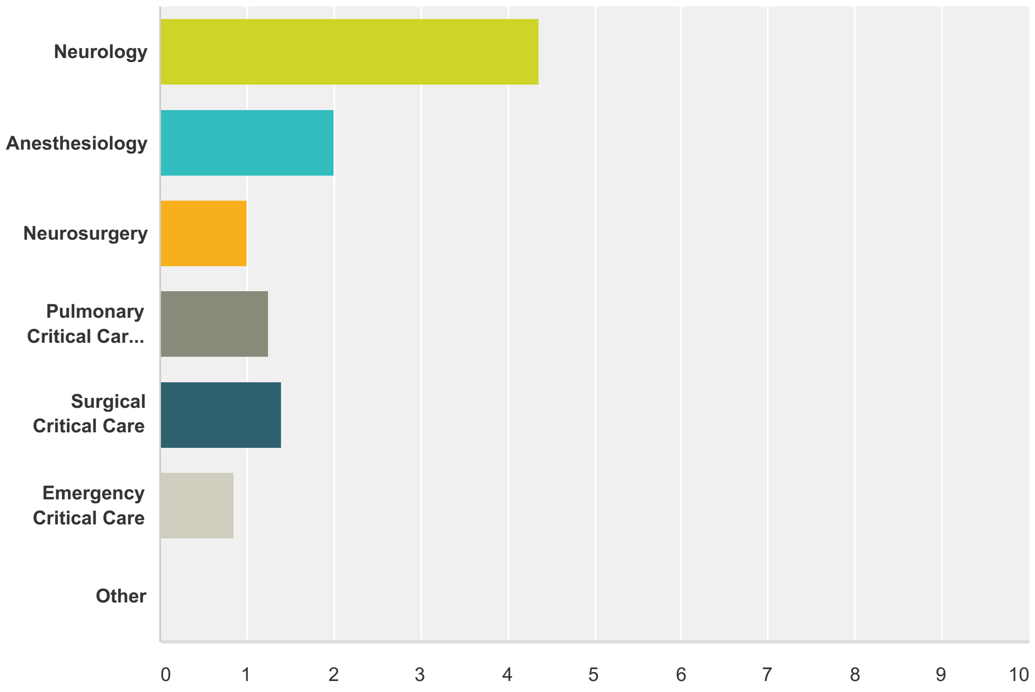

Answer Choices	Average Number	Total Number	Responses
Neurology	4	118	27
Anesthesiology	2	26	13
Neurosurgery	1	6	6
Pulmonary Critical Care / Internal Medicine	1	10	8
Surgical Critical Care	1	7	5
Emergency Critical Care	1	6	7
Other	0	0	1
Total Respondents: 27			

Q38 At your institution, how many intensive care units do the neurocritical care faculty and fellows cover?

Answered: 28 Skipped: 5

#	Responses	Date
1	1	7/22/2016 1:06 PM
2	2	7/22/2016 10:43 AM
3	1	7/22/2016 8:22 AM
4	1	7/21/2016 12:50 PM
5	1	7/21/2016 9:21 AM
6	2	7/21/2016 3:56 AM
7	1	7/20/2016 5:14 PM
8	1	7/20/2016 3:42 PM
9	2	7/20/2016 3:18 PM
10	1	7/20/2016 2:40 PM
11	1	7/20/2016 1:26 PM
12	2	7/20/2016 9:42 AM
13	3	7/19/2016 1:02 PM
14	1	7/15/2016 6:23 PM
15	1	7/15/2016 7:03 AM
16	1	7/14/2016 3:47 PM
17	1	7/14/2016 11:10 AM
18	2	7/13/2016 10:14 PM
19	2	7/13/2016 9:48 PM
20	2	7/13/2016 8:12 PM
21	1	7/13/2016 6:05 PM
22	2	7/13/2016 5:57 PM
23	5	7/13/2016 5:26 PM
24	1	7/13/2016 5:23 PM
25	7	7/13/2016 5:05 PM
26	1	7/13/2016 4:41 PM
27	2	7/13/2016 4:33 PM
28	2	6/22/2016 11:22 AM

Q39 At your institution, how many total beds do the neurocritical care faculty and fellows cover (please write in)?

Answered: 28 Skipped: 5

#	Responses	Date
1	20	7/22/2016 1:06 PM
2	36	7/22/2016 10:43 AM
3	12 with overflow capacity	7/22/2016 8:22 AM
4	16	7/21/2016 12:50 PM
5	14	7/21/2016 9:21 AM
6	20	7/21/2016 3:56 AM
7	23	7/20/2016 5:14 PM
8	23	7/20/2016 3:42 PM
9	22	7/20/2016 3:18 PM
10	16+ (flow over to other units)	7/20/2016 2:40 PM
11	17-28	7/20/2016 1:26 PM
12	32	7/20/2016 9:42 AM
13	64	7/19/2016 1:02 PM
14	14-28	7/15/2016 6:23 PM
15	8	7/15/2016 7:03 AM
16	24	7/14/2016 3:47 PM
17	20	7/14/2016 11:10 AM
18	30	7/13/2016 10:14 PM
19	36	7/13/2016 9:48 PM
20	32	7/13/2016 8:12 PM
21	15	7/13/2016 6:05 PM
22	32	7/13/2016 5:57 PM
23	54	7/13/2016 5:26 PM
24	16	7/13/2016 5:23 PM
25	20	7/13/2016 5:05 PM
26	13	7/13/2016 4:41 PM
27	20	7/13/2016 4:33 PM
28	32	6/22/2016 11:22 AM

Q40 Are the units considered "open" or "closed"? In the "Other" box, please describe the structure if neither "open" nor "closed" as defined below applies.

Answered: 25 Skipped: 8

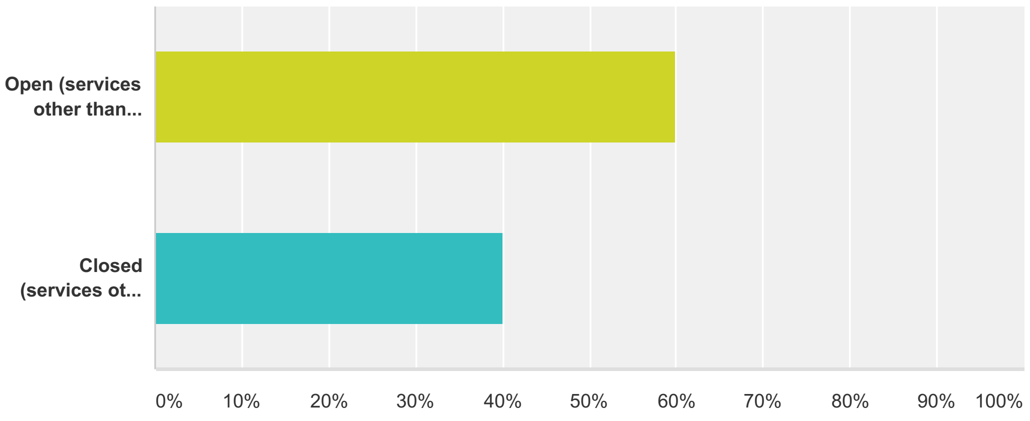

Answer Choices	Responses	
Open (services other than Neurocritical Care can admit patients and enter orders)	**60.00%**	15
Closed (services other than Neurocritical Care can NOT admit patients and enter orders)	**40.00%**	10
**Total**		**25**

Q41 Is there a "step-down" unit at your institution where the neurocritical care faculty and fellows care for patients?

Answered: 28 Skipped: 5

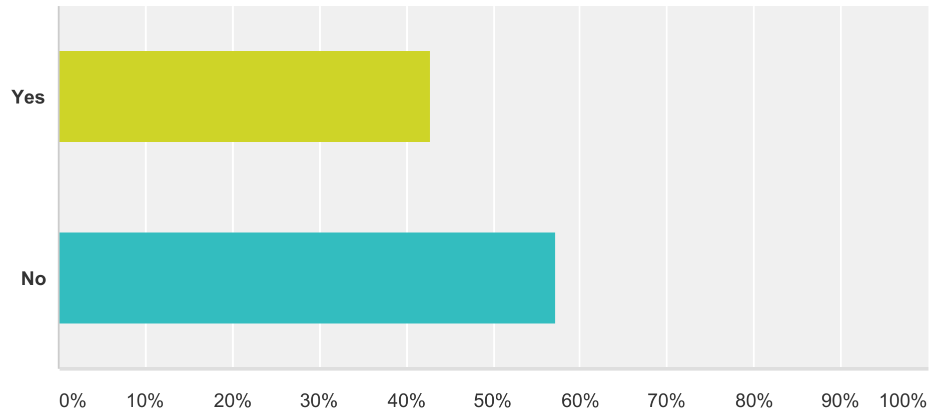

Answer Choices	Responses	
Yes	**42.86%**	12
No	**57.14%**	16
**Total**		**28**

Q42 Do the neurocritical care faculty and fellows provide consultations on other units in your institution?

Answered: 28 Skipped: 5

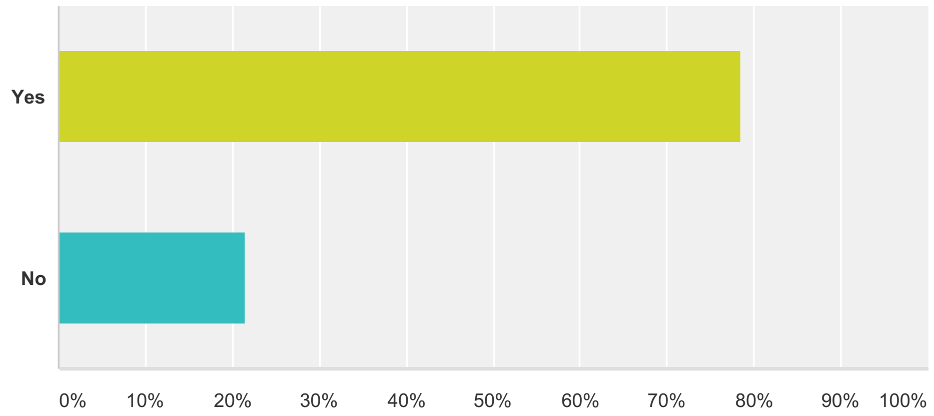

Answer Choices	Responses	
Yes	**78.57%**	22
No	**21.43%**	6
**Total**		**28**

Q43 Do residents provide coverage in your ICU(s)?

Answered: 28 Skipped: 5

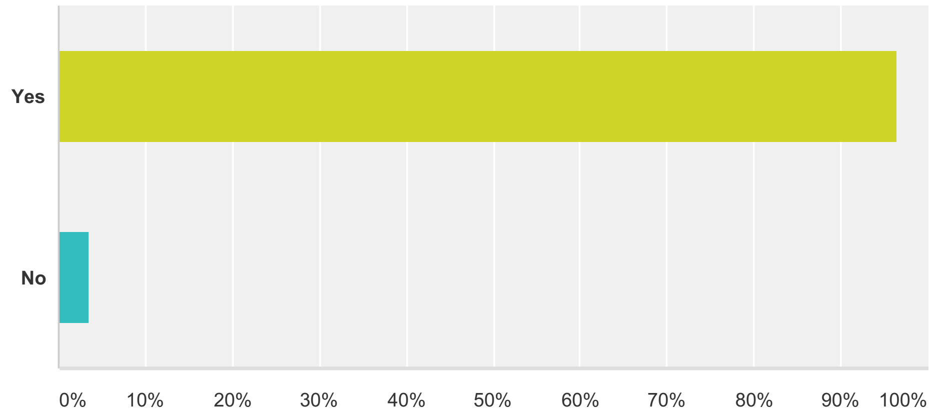

Answer Choices	Responses	
Yes	**96.43%**	27
No	**3.57%**	1
**Total**		**28**

Q44 Do advanced practice nurses (APN) or physician assistants (PA) provide coverage in your ICU(s)?

Answered: 26 Skipped: 7

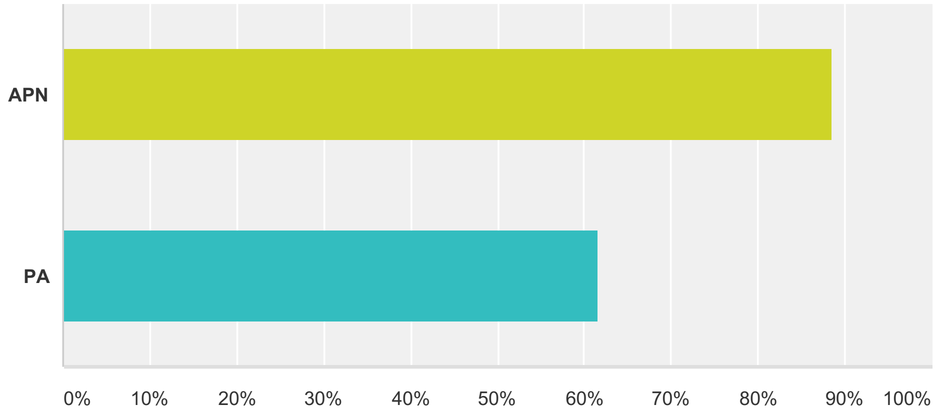

Answer Choices	Responses	
APN	**88.46%**	23
PA	**61.54%**	16
**Total Respondents: 26**		

Q45 With respect to staffing, is there 24/7 in-hospital physician or advanced practice provider coverage dedicated to your ICU(s)?

Answered: 28 Skipped: 5

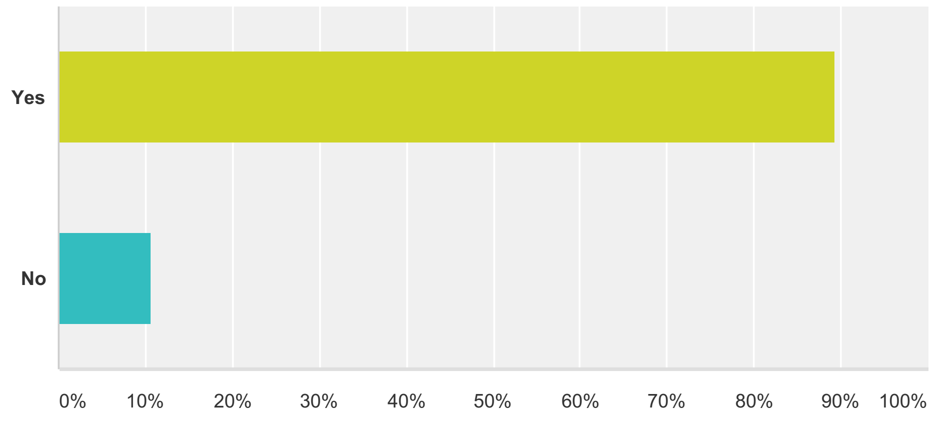

Answer Choices	Responses	
Yes	**89.29%**	25
No	**10.71%**	3
**Total**		**28**

Q46 If there is dedicated 24/7 provider coverage in your ICU, who are the providers involved in night-time in-hospital coverage (please check ll that apply)?

Answered: 25 Skipped: 8

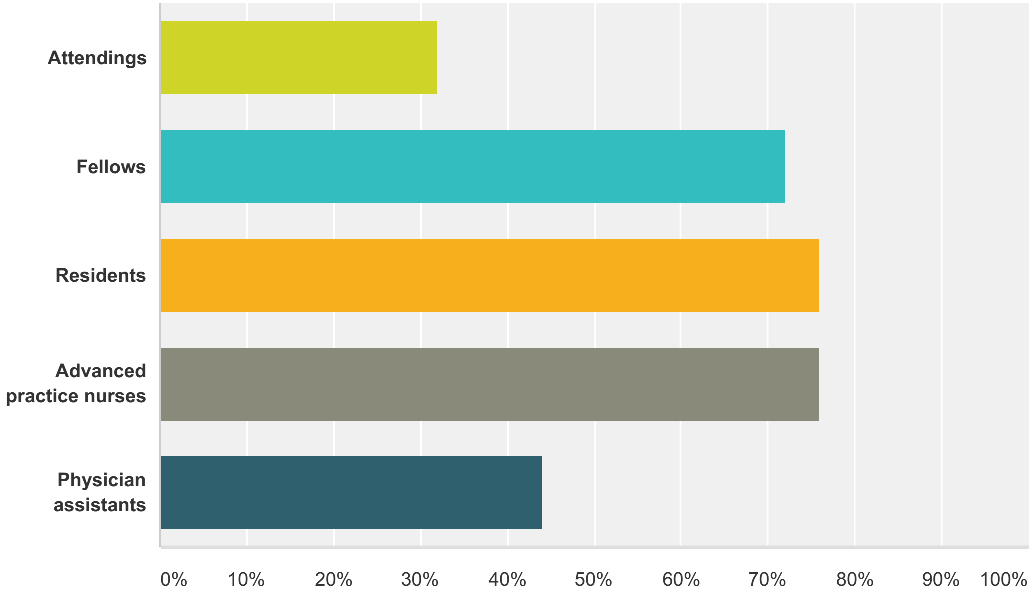

Answer Choices	Responses	
Attendings	32.00%	8
Fellows	72.00%	18
Residents	76.00%	19
Advanced practice nurses	76.00%	19
Physician assistants	44.00%	11
Total Respondents: 25		
